# Inhibitory inputs from thalamus promote resilient spiking in tail of striatum

**DOI:** 10.1016/j.isci.2025.113880

**Published:** 2025-10-27

**Authors:** Laura M. Haetzel, Jan Gründemann

**Affiliations:** 1 German Center for Neurodegenerative Diseases (DZNE), Venusberg-Campus 1/99, 53127 Bonn, Germany; 2 University of Bonn, Faculty of Medicine, Venusberg-Campus 1/33, 53127 Bonn, Germany; 3 Boehringer Ingelheim Fonds, Stiftung für Medizinische Grundlagenforschung, Schusterstr. 46-48, 55116 Mainz, Germany

**Keywords:** Neuroscience, Sensory neuroscience

## Abstract

Thalamic afferents form glutamatergic synapses with striatal medium spiny neurons (MSNs). These projections are involved in complex learning processes and have primarily been studied in rostral striatum. Caudal regions, such as tail of striatum (TS), have only recently gained attention. Medial geniculate body (auditory thalamus; MGB) provides functional excitatory inputs to MSNs in TS; however, it is unclear how MGB regulates striatal output signals. We therefore performed optogenetics-assisted circuit mapping in *ex vivo* brain slices to examine how MGB modulates neuronal output in TS MSNs. Whole-cell recordings revealed that activation of MGB terminals increases MSN firing specifically at high levels of postsynaptic depolarization. In addition to the well-described excitatory thalamostriatal projections, we identified a sparse population of GABAergic projection neurons in higher-order MGB that inhibit TS MSNs through a GABA_B_-receptor-mediated mechanism. Together, these results provide a conceptual framework for MGB-driven state-dependent modulation of striatal output signals.

## Highlights

• MGB activation gives rise to both excitatory and inhibitory responses in TS MSNs
• Hyperpolarizing MGB projections extend the dynamic range of MSN neuronal output
• Modulation of firing dynamics is dictated by depolarization state and MSN cell type
• Long-range GABAergic neurons from MGB hyperpolarize MSNs via GABA_B_ receptors

## Graphical abstract

**Figure:**
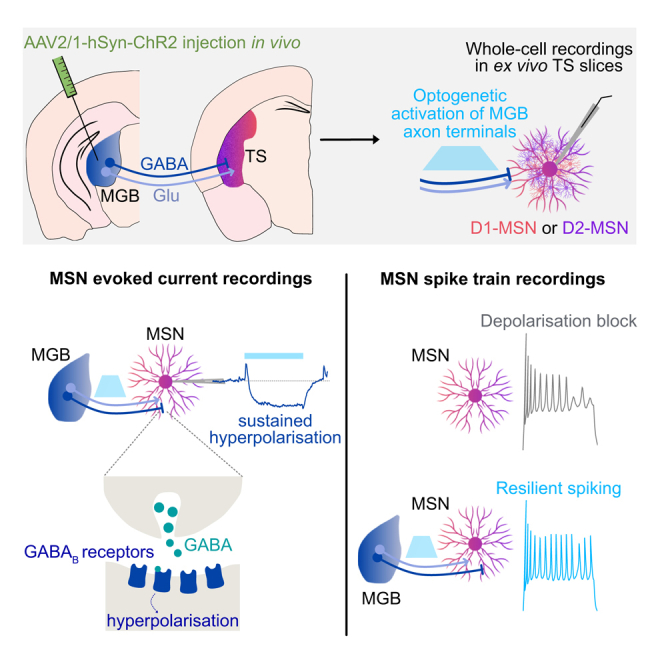

## Introduction

Integrating sensory information to guide behavior is a complex process that relies on intricately weighted synapses. A key element of this integration is selecting salient stimuli from a crowded sensory environment. This function, also known as sensory gating, is primarily attributed to thalamic circuitry.^1^ Thalamic neurons form synaptic contacts with various brain regions including medium spiny neurons (MSNs) in the striatum. While thalamostriatal synapses have not been as extensively studied as their corticostriatal counterparts, they have been implicated in discrimination learning, attention shifting, and behavioral flexibility.^2^^,^^3^^,^^4^^,^^5^^,^^6^^,^^7^

The properties of thalamostriatal synapses differ substantially across thalamic nuclei.^8^^,^^9^^,^^10^ Within the intralaminar thalamus alone, central lateral and parafascicular thalamostriatal neurons are distinct in their physiological features and postsynaptic targets. While central lateral neurons rely on low-threshold calcium channels to fire in burst patterns, parafascicular neurons fire action potentials (APs) at a low frequency. Postsynaptically, central lateral neurons terminate onto dendritic spines of MSNs, while parafascicular neurons predominantly form synapses onto dendritic shafts.^2^^,^^10^ In addition to innervating MSNs, select populations of thalamic neurons can modulate striatal cholinergic interneurons, suppress release from glutamatergic terminals through presynaptic inhibition, and even trigger striatal dopamine release.^2^^,^^11^^,^^12^^,^^13^ The properties of thalamostriatal synapses are therefore heterogeneous and must be assessed separately by nucleus of origin.

Medial geniculate body (MGB, auditory thalamus) is embedded in the ascending auditory pathway and sends a dense projection to tail of striatum (TS).^14^^,^^15^^,^^16^ Though considered a classical relay structure, MGB neurons are modulated by behavioral state, display heterogeneous plasticity patterns, and are necessary for appetitive and fear learning.^17^^,^^18^^,^^19^^,^^20^^,^^21^ Particularly higher-order MGB neurons are therefore thought to encode context-dependent representations of sound and convey these to downstream regions including amygdala and TS to guide behavior.^15^^,^^17^

TS and thalamostriatal projections to TS have only recently gained attention.^14^^,^^16^ TS is located at the caudal end of striatum and is a functional subdomain implicated in threat detection and auditory discrimination.^3^^,^^22^^,^^23^^,^^24^ Like other striatal regions, TS receives major glutamatergic inputs from cortex and thalamus.^15^^,^^25^^,^^26^ However, TS exhibits striking differences in cellular architecture when compared to anterior striatum. In general, the striatum contains two molecularly defined subtypes of MSNs: MSNs that either express dopamine receptor subtype 1 (D1-MSNs) or subtype 2 (D2-MSNs).^27^^,^^28^ While D1-MSNs and D2-MSNs intermingle to form a mosaic across the anterior striatum, TS exhibits a non-random distribution of MSN subtypes that is characterized by three clusters: the D1/D2 intermingled domain, the D1/D2 co-expressing domain, and the D2-lacking domain.^29^^,^^30^ Up to 33% of TS MSNs in the co-expressing domain are positive for both D1 and D2 receptor subtypes, suggesting that the conventional definition of striatal cell types may not fully capture MSN subtypes in TS.^31^ Together, this illustrates that TS has a distinctive cellular composition that likely translates to unique physiological and circuit function.

TS MSNs not only represent auditory information but also support more complex functions, such as attention and sensory-guided learning.^3^^,^^6^^,^^7^^,^^14^^,^^22^ These functions are likely supported by thalamostriatal inputs from MGB. Functional connectivity between thalamic neurons from MGB to MSNs in TS has been conclusively established. Optogenetic activation of MGB axons in *ex vivo* slices leads to both excitatory postsynaptic currents and inhibitory postsynaptic currents in TS MSNs.^14^^,^^16^ MGB neurons were additionally found to innervate parvalbumin-positive (PV) interneurons in TS, driving cell type-specific feedforward inhibition preferentially onto D2-MSNs.^14^^,^^16^

However, it remains unclear how MGB projections regulate TS output. Here, we investigated how MGB afferents modulate AP firing in TS MSNs. Optogenetics-assisted circuit mapping experiments in *ex vivo* brain slices showed that MGB modulates rate coding in a depolarization state-dependent manner. Modulation of temporal coding was also determined by depolarization state and additionally influenced by MSN subtype. Finally, optogenetic activation of MGB terminals led to inhibitory responses in MSNs that were both monosynaptic and GABA_B_ receptor mediated. Conditional retrograde tracing in *VGAT*-Cre mice revealed a sparse population of GABAergic neurons projecting from MGB to TS. Together, these results suggest that parallel excitatory and inhibitory projections from MGB shape striatal output by promoting resilient spiking in TS MSNs.

## Results

### MGB projects to TS

**Figure 1:**
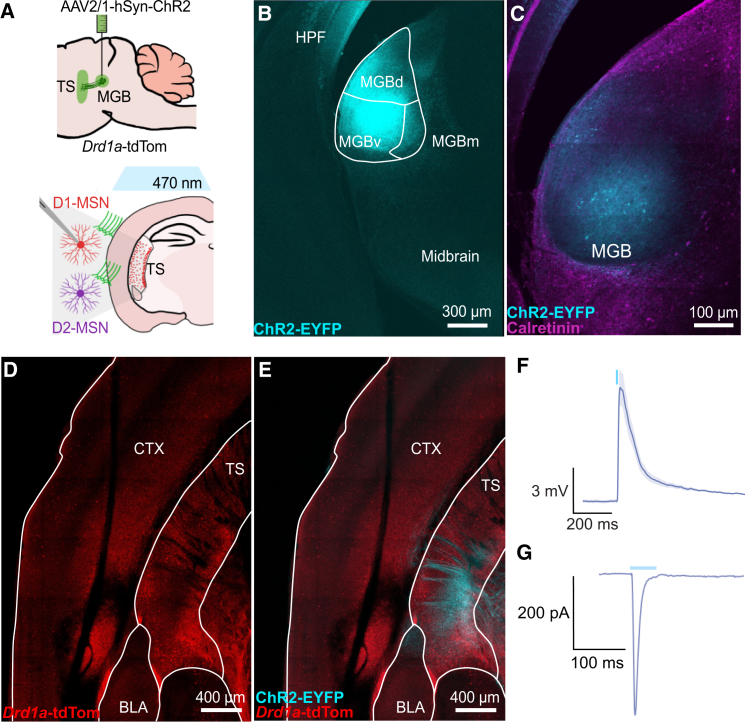
MGB projects to tail of striatum (A) Experimental strategy for optogenetic activation of thalamostriatal terminals. (B and C) (B) Representative image confirming the injection site (scale bar: 300 μm) (C) counterstained for calretinin to verify all ChR2-positive cells were located within MGB (scale bar: 100 μm). (D and E) (D) Representative coronal slice recovered and fixed post-*ex vivo* recording showing tail of striatum from *Drd1a*-tdTomato mice with (E) ChR2-positive axons (scale bars: 400 μm). (F) Current clamp recordings of excitatory response to optogenetic stimulation (10 ms; 470 nm light pulse indicated by blue line) in MSNs (represented as mean trace of 48 MSNs±SEM). (G) Voltage-clamp recording of an EPSC in an MSN after optogenetic activation (50 ms; light pulse indicated by blue line) of MGB (example trace from one representative MSN. Holding potential: −70 mV). Atlas overlays generated with the Allen Mouse Brain Reference Atlas.^32^

We injected an adeno-associated virus containing channelrhodopsin (AAV-ChR2-EYFP) into MGB of *Drd1a*-tdTomato mice to record optogenetically evoked postsynaptic potentials (oPSPs) in MSNs (Figure 1A). Region-specific expression of ChR2 was confirmed with an antibody stain against calretinin to delineate the margins of MGB (Figures 1B and 1C). ChR2-positive MGB axons were found in TS but not anterior striatal regions (Figures 1D, 1E, and S1A–S1C). In agreement with previous studies, whole-cell recordings of MSNs in TS showed excitatory oPSPs in current clamp recordings and excitatory inward currents in a representative voltage clamp recording (Figures 1F, 1G, and S1D–S1F).^14^^,^^16^

### Thalamostriatal modulation promotes resilient spiking in TS MSNs

Next, we assessed the effect of MGB activation on AP firing in TS. We evoked spike trains in MSNs by applying depolarizing current steps (500 ms) until the MSN entered depolarization block. As MSNs rarely cease AP firing entirely, the threshold for depolarization block is generally set using reductions in AP number or height as metrics.^33^ Here, we defined depolarization block as a height reduction in at least half the APs in the spike train (see STAR Methods; quantification and statistical analysis). The current step at which half the APs were reduced in height was defined individually for each MSN.

**Figure 2:**
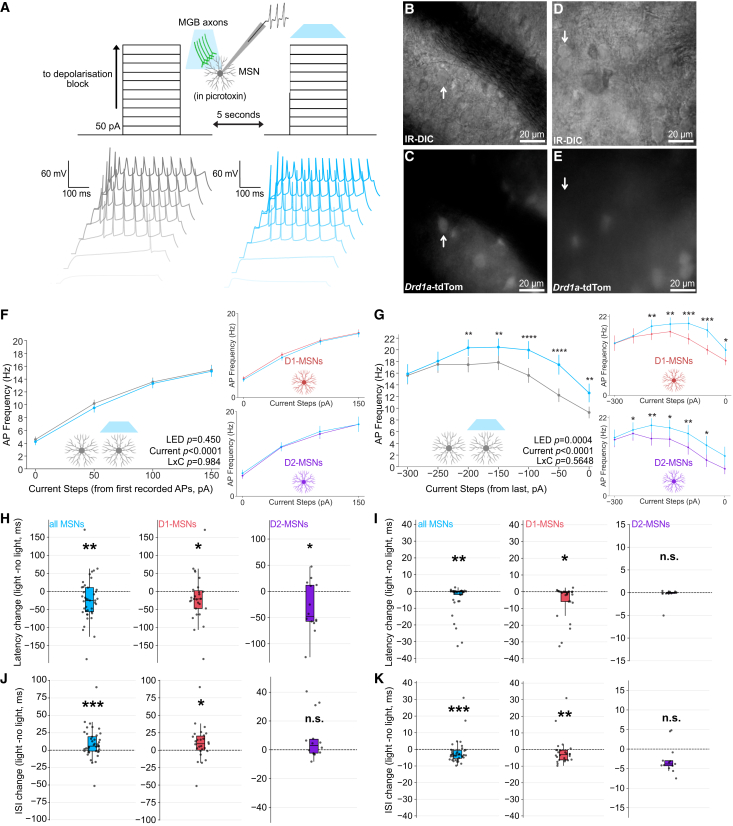
Thalamostriatal modulation promotes resilient spiking in TS MSNs (A) Experimental strategy for current injections with simultaneous optogenetic stimulation (top) and spike trains from a representative MSN (bottom) recorded first without (gray) and then with (blue) optogenetic activation of MGB terminals. (B–E) (B) Simultaneous infrared differential interference contrast-based imaging (IR-DIC) and (C) epifluorescence microscopy to identify *Drd1a*-tdTom-positive (D1-MSNs) and (D and E) *Drd1a*-tdTom-negative (putative D2-MSNs) cells in *Drd1a*-tdTom mice (scale bars: 20 μm). (F) Input-output curves for mean AP frequency normalized to current step with first recorded spikes for the no light (gray) and light (blue) conditions for all MSNs (*n* = 39 cells from 20 slices across 8 mice; represented as mean ± SEM), D1-MSNs (top inset; *n* = 25 cells from 17 slices across 8 mice; mean ± SEM), and D2-MSNs (bottom inset; *n* = 14 cells from 12 slices across 6 mice; mean ± SEM). (G) Input-output curves for mean AP frequency normalized to highest recorded current injection for all MSNs (*n* = 35 cells from 20 slices across 8 mice; mean ± SEM; two-way ANOVA; LED *p* = 0.0003), D1-MSNs (top inset; *n* = 22 cells from 17 slices across 8 mice; mean ± SEM; two-way ANOVA; LED *p* = 0.0082), and D2-MSNs (bottom inset; *n* = 13 cells from 12 slices across 6 mice; mean ± SEM; two-way ANOVA; LED *p* = 0.0137; *post hoc* Wilcoxon signed-rank test; ∗*p* < 0.05, ∗∗*p* < 0.01, ∗∗∗*p* < 0.001, and ∗∗∗∗*p* < 0.0001). (H) Change in latency to first action potential during early current steps in all MSNs (left; *n* = 39 cells from 20 slices across 8 mice; data represented as a standard boxplot with mean and interquartile range; Wilcoxon signed-rank test, *p* = 0.0057 and ∗∗*p* < 0.01), D1-MSNs (middle; *n* = 25 cells from 17 slices across 8 mice; represented as mean with interquartile range; Wilcoxon signed-rank test; *p* = 0.0626 and ∗*p* < 0.05), and D2-MSNs (right; *n* = 14 cells from 12 slices across 6 mice; Wilcoxon signed-rank test; *p* = 0.0295 and ∗*p* < 0.05). (I) Change in latency to first action potential during late current steps in all MSNs (left; *n=*38 cells from 20 slices across 8 mice; represented as mean with interquartile range; Wilcoxon signed-rank test; *p* = 0.0120 and ∗∗*p* < 0.01), D1-MSNs (middle; *n* = 25 cells from 17 slices across 8 mice, represented as mean with interquartile range; Wilcoxon signed-rank test; *p* = 0.0160 and ∗*p* < 0.05), and D2-MSNs (right; *n* = 13 cells from 12 slices across 6 mice, represented as mean with interquartile range; Wilcoxon signed-rank test; *p* = 0.7869). (J) Change in inter-spike interval (ISI) during early current steps in all MSNs (left; *n* = 39 cells from 20 slices across 8 mice; represented as mean with interquartile range; Wilcoxon signed-rank test; *p* = 0.0007 and ∗∗∗*p* < 0.001), D1-MSNs (middle); *n* = 25 cells from 17 slices across 8 mice; represented as mean with interquartile range; Wilcoxon signed-rank test; *p* = 0.0051 and ∗∗*p* < 0.01), and D2-MSNs (right; *n* = 14 cells from 12 slices across 6 mice; *p* = 0.1726, Wilcoxon signed-rank test). (K) Change in ISI during late current steps in all MSNs (left; *n* = 38 cells from 20 slices across 8 mice; represented as mean with interquartile range; Wilcoxon signed-rank test; *p* = 0.0006 and ∗∗∗*p* < 0.001), D1-MSNs (middle; *n* = 25 cells from 17 slices across 8 mice; represented as mean with interquartile range; Wilcoxon signed-rank test; *p* = 0.0012 and ∗∗*p* < 0.01), and D2-MSNs (right; *n* = 13 cells from 12 slices across 6 mice; represented as mean with interquartile range; Wilcoxon signed-rank test; *p* = 0.0681).

MSN spike trains were recorded using successively increasing current injections at 50 pA increments. After each current step, the same amount of depolarizing current was injected with optogenetic activation of MGB terminals for direct comparison (Figure 2A). MSNs were analyzed cumulatively and by subtype (D1-MSNs vs. D2-MSNs). D1-MSNs and D2-MSNs were distinguished during recording by transgenic *Drd1a*-tdTomato expression (Figures 2B–2E).

MGB modulation of MSN APs differed by depolarization state. During early current injections (low depolarization), optogenetic activation of MGB did not affect AP frequency in either MSN subtype (Figure 2F; see Figures S2A and S2B for current steps without normalization). Conversely, MGB activation significantly increased AP frequency in both MSN subtypes during late current injections (high depolarization; Figure 2G; LED *p* = 0.0003).

Given that MGB strongly modulates AP frequency in MSNs, we investigated AP properties in more detail. Overall, MGB activation broadly affected MSN AP dynamics across cell types and depolarization states, with a particularly prominent effect on width and rise time (Figures S3A–S3F; Tables S2 and S3). To specifically examine MGB modulation of temporal coding, we focused on latency to first spike and inter-spike interval (ISI). During low depolarization states, latency to first spike was decreased in all MSNs and D2-MSNs, with a strong trend toward reduction in D1-MSNs (Figures 2H and S3A–S3C; all MSNs *p* = 0.0057, D1-MSNs *p* = 0.0626, and D2-MSNs *p* = 0.0295). For D1-MSNs, latency was significantly reduced during high depolarization states, whereas latency in D2-MSNs was no longer affected (Figures 2I and S3D–S3F; D1-MSNS *p* = 0.0160 and D2-MSNS *p* = 0.7869). This is likely explained by low baseline spike latency observed in D2-MSNs over D1-MSNs (Figures S3E and S3F).

ISI was oppositely affected depending on depolarization state. During low depolarization, ISI was increased in D1-MSNs (Figures 2J and S3A–S3C; all MSNs *p* = 0.0007 and D1-MSNs *p* = 0.0051) but decreased during high depolarization (Figures 2J, 2K, and S3D–S3F; all MSNs *p* = 0.0006 and D1-MSNs *p* = 0.0012). ISI in D2-MSNs was not strongly modulated but showed similar trends to D1-MSNs in both depolarization conditions (Figures 2J, 2K, and S3D–S3F).

### Activation of MGB terminals evokes both excitatory and inhibitory changes in membrane potential in TS MSNs

**Figure 3:**
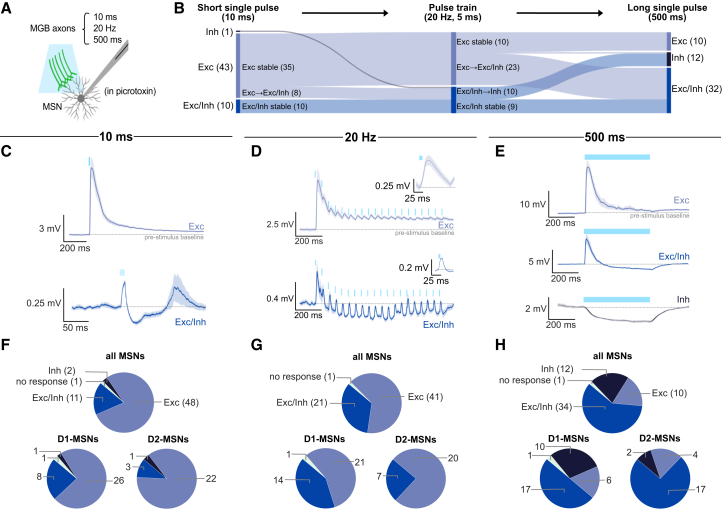
Activation of MGB terminals evokes both excitatory and inhibitory activity in TS MSNs (A) Schematic illustrating approach for recording MSN responses to MGB axon activation using a 10 ms single pulse, a 20 Hz pulse train, and a 500 ms single pulse. (B) Sankey diagram showing changing responses from the same MSNs to 10 ms, 20 Hz, and 500 ms light pulses (*n* = 54 cells from 24 slices across 12 mice). (C) Average of all excitatory responses (light blue, mean of 48 cells ± SEM) and mixed excitatory/inhibitory responses (blue; mean of 11 cells ± SEM) elicited by a short 10 ms light pulse. (D) Average of all excitatory responses (light blue, mean of 41 cells ± SEM) and mixed responses (blue, mean of 21 cells ± SEM) to a 20 Hz pulse train. (E) Average of all excitatory (light blue, mean of 10 cells ± SEM), mixed (blue; average of 34 cells ± SEM), and inhibitory (dark blue, mean of 12 cells ± SEM) responses to a long 500 ms light pulse. (F) Breakdown of response types to a 10 ms light pulse for all MSNs (*n* = 62 cells from 24 slices across 15 mice; 77% excitation, 18% mixed, and 3% inhibition), D1-MSNs (*n* = 36 cells from 24 slices across 15 mice; 72% excitation, 22% mixed, and 3% inhibition), and D2-MSNs (*n* = 26 cells from 23 slices across 14 mice; 85% excitation, 11% mixed, and 4% inhibition). (G) Breakdown of response types in response to a 20 Hz pulse train for all MSNs (*n* = 63 cells from 24 slices across 15 mice; 65% excitation and 33% mixed), D1-MSNs (*n* = 36 cells from 24 slices across 15 mice; 58% excitation and 39% mixed), and D2-MSNs (*n=*27 cells from 23 slices across 14 mice, 74% excitation and 26% mixed). (H) Breakdown of response types to a 500 ms light pulse for all MSNs (*n* = 57 cells from 23 slices across 12 mice; 18% excitation, 60% mixed, 21% and inhibition), D1-MSNs (*n* = 34 cells from 22 slices across 12 mice; 18% excitation, 50% mixed, and 29% inhibition), and D2-MSNs (*n* = 23 cells from 17 slices across 10 mice; 17% excitation, 74% mixed, and 9% inhibition).

During high depolarization states, MSNs enter a depolarization block during which AP firing is diminished due to reduced recovery from voltage-dependent sodium channel inactivation. Our results show that optogenetic activation maintains peak firing frequency during large current injections, suggesting that MGB may prevent depolarisation-induced AP reduction by lowering membrane potential to aid recovering from Na^+^ channel inactivation and thus promote resilient spiking in MSNs. To further examine the effects of MGB on MSN membrane potential, we assessed oPSPs across multiple stimulation parameters: short pulse (10 ms), pulse train (5 ms; 20 Hz), and long pulse (500 ms; Figure 3A). Stimulation of MGB at various frequencies (1, 5, and 10 Hz) revealed similar response profiles in MSNs (Figure S4).

Excitatory and mixed excitatory/inhibitory responses were recorded across all three stimulation parameters (Figures 3B–3E). Activation of MGB afferents with a short light pulse primarily evoked excitatory responses (Figure 3F; 77% excitation, 18% mixed, and 3% inhibition; *n* = 63 cells). Stimulation with a 20 Hz pulse train evoked a higher proportion of mixed excitatory/inhibitory responses (Figure 3G; 65% excitation and 33% mixed; *n* = 63 cells). The response distributions of all MSNs to the short pulse versus the pulse train were not significantly different (Table S1; *p* = 0.1289).

**Figure 4:**
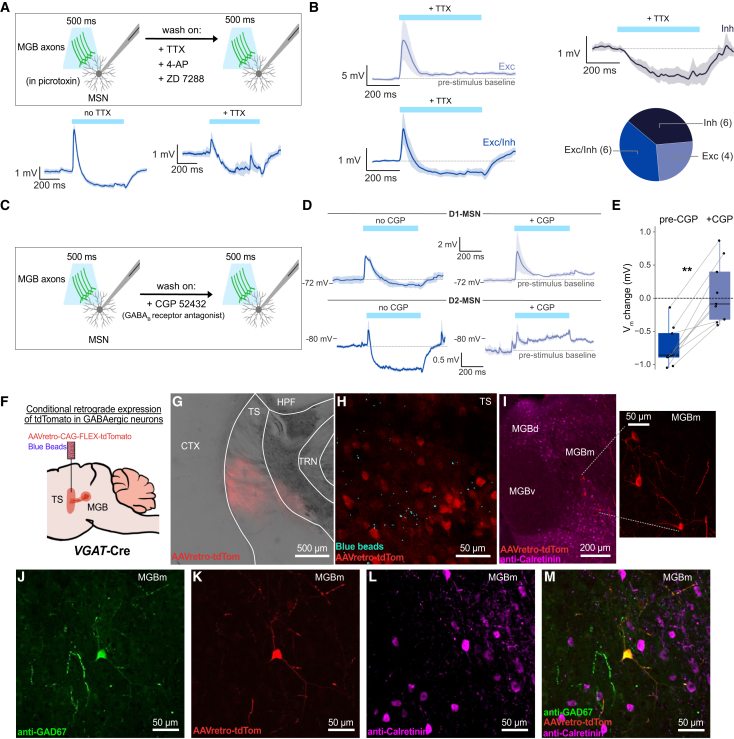
Long-range GABAergic neurons from MGB inhibit TS MSNs via GABA_B_ receptors (A) Experimental strategy to isolate monosynaptic inputs via wash-on of TTX, 4-AP, and ZD 7288 (top). Example D1-MSN responding to a 500 ms light pulse before (bottom left) and after (bottom right) wash-on (mean of 5 sweeps ± SEM). (B) Different types of monosynaptic responses recorded in MSNs following 500 ms optogenetic activation of MGB axons with average traces and breakdown of response types (bottom right; *n* = 16 cells from 16 slices across 8 mice, including 6 D1-MSNs and 10 D2-MSNs, 25% excitation, 38% mixed, and 38% inhibition). (C) Pharmacological blockade of monosynaptic GABA_B_-receptor mediated responses via wash-on of CGP 52432. (D) Response of one example D1-MSN prior to (top left, mean of 5 sweeps±SEM) and after (top right, mean of 5 sweeps ± SEM) GABA_B_ receptor blockade and one example D2-MSN prior to (bottom left, mean of 5 sweeps ± SEM) and after (bottom right, mean of 5 sweeps ± SEM) GABA_B_ receptor blockade. (E) Change in membrane potential (V_m_) from pre-stimulation baseline before and after GABA_B_ receptor blockade (*n* = 9 cells from 9 slices across 4 mice including 5 D1-MSNs and 4 D2-MSNs, data represented as a standard boxplot with mean and interquartile range; Wilcoxon signed-rank test; *p* = 0.002 and ∗∗*p* < 0.01; change from baseline measured as average V_m_ 200–300 ms after light pulse onset). (F) Injection of Cre-dependent retrograde virus carrying tdTomato into TS of *VGAT*-Cre mice. (G and H) (G) Confirmation of injection in TS with (H) co-injection of synthetic blue beads (scale bars G: 500 μm, H: 50 μm). (I) tdTomato-positive cell bodies in medial MGB resulting from injection of retrograde virus in TS (scale bars: 200 μm, 50 μm [inset]). (J–M) GAD67 counterstaining to confirm that tdTomato-positive cell bodies within MGB are GABAergic (scale bars J–M: 50 μm). Atlas overlays generated with the Allen Mouse Brain Reference Atlas.^32^

Conversely, MSN responses to the long pulse significantly differed from responses to the short pulse (*p* < 0.0001) and pulse train (*p* < 0.0001). The long pulse primarily evoked mixed excitatory/inhibitory responses and inhibitory responses (Figure 3H; 18% excitation, 60% mixed, and 21% inhibition; *n* = 57 cells). Cells that displayed inhibitory responses to the long pulse responded with mixed excitation/inhibition to the pulse train, suggesting that long-pulse activation is needed to reveal inhibitory responses (Figure 3B). We therefore proceeded with the long pulse to assess the mechanistic features of MGB-driven inhibition of TS MSNs (Figure 4).

### Long-range GABAergic neurons from MGB inhibit TS MSNs via GABA_B_ receptors

MSN inhibition is largely driven by local striatal interneurons.^34^^,^^35^ As our recordings were conducted in the presence of picrotoxin, local interneurons were ruled out as a source of inhibitory oPSPs. Instead, we investigated the hypothesis that inhibitory postsynaptic potentials (IPSPs) are directly driven by MGB inputs, given that long-range inhibitory inputs to striatum have been identified in the cortex.^36^^,^^37^^,^^38^^,^^39^

To isolate monosynaptic responses, we repeated the recordings in tetrodotoxin (TTX) to block sodium channels, 4-aminopyridine (4-AP) to block potassium channels, and ZD 7288 to block hyperpolarization-activated currents (Figures 4A and 4B; *n* = 16 cells).^40^ We activated MGB terminals with 500 ms light pulses and observed all three response types in MSNs in the presence of TTX, 4-AP, and ZD 7288 (25% excitation, 38% mixed, and 38% inhibition; Figures 4B and 4C). This result points toward a direct inhibitory projection from MGB to TS.

Given that our recordings were performed in picrotoxin, GABA_A_ receptors were pharmacologically blocked, and we therefore asked if GABA_B_ receptors were involved in MGB-driven inhibition of TS MSNs. Metabotropic GABA_B_ receptors are known to mediate synaptic events occurring across hundreds of milliseconds, similar to those observed in our recordings (Figures 4A and 4B).^41^^,^^42^^,^^43^ To test for the role of GABA_B_ receptors, we recorded monosynaptic inhibitory currents and subsequently washed on the highly competitive GABA_B_ receptor antagonist CGP 52432 (Figure 4C).^44^ Indeed, we saw a significant reduction in TS MSN inhibition when optogenetically activating MGB afferents after CGP 52432 wash-on, suggesting that GABA_B_ receptors play a key role in MGB-TS-driven hyperpolarization of MSNs (Figures 4D and 4E; *n* = 9 cells, *p* = 0.002).

Monosynaptic inhibition of TS MSNs implies the presence of long-range GABAergic neurons projecting from MGB to TS. To confirm this anatomically, we injected a retrograde AAV carrying Cre-dependent tdTomato into TS of *VGAT*-Cre mice (Figures 4F–4H). This revealed a sparse population of long-range inhibitory neurons that were predominantly located in medial MGB (MGBm; Figures 4I and S7A–S7L). These neurons were furthermore positive for the inhibitory marker GAD67, thus confirming their identity as long-range GABAergic neurons (Figures 4J–4M and S7M–S7X).

## Discussion

Using optogenetics-assisted *ex vivo* circuit mapping, we explored the modulatory properties of projections from MGB to TS (Figure 1). We found that MGB sends parallel excitatory and inhibitory projections specifically to TS and regulates MSN signals in a depolarization state- and cell type-dependent manner.^14^^,^^16^^,^^26^^,^^30^ This projection was not only anatomically precise but also highly reliable, producing responses in nearly all recorded MSNs (Figure 3).

While excitatory inputs from MGB to TS have been previously described, the effect of these neurons on MSN AP firing is less well understood.^11^^,^^14^^,^^16^^,^^45^ *In vivo*, MSNs receive a convergence of glutamatergic inputs to enter an “up state” from which they can fire APs.^27^^,^^46^^,^^47^ Since this state cannot be induced in *ex vivo* slice preparations, we modeled convergent excitation onto MSNs by combining somatic depolarization with optogenetic activation of MGB afferents (Figure 2A). This revealed depolarization state-dependent modulation of firing rate (Figures 2F and 2G). MSNs exhibit opposite responses to GABA at hyperpolarized versus subthreshold membrane potentials due to shifts in receptor activity.^48^ Therefore, it is plausible that different postsynaptic receptors in MSNs drive depolarization-state-dependent responses to MGB inputs.

In addition to rate coding, MGB broadly affects AP dynamics and temporal coding in MSNs (Figure S3F; Tables S2 and S3). Width and rise time were significantly reduced across cell types and depolarization states. Conversely, modulation of latency to first spike was cell type specific (Figures 2H and 2I; Tables S2 and S3). This is in line with a large body of literature showing that MSN subtypes have distinctive physiological properties due to differing developmental trajectories, molecular profiles, and projection targets.^27^^,^^49^^,^^50^^,^^51^^,^^52^

Both *ex vivo* and *in vivo* recordings have shown physiological differences between D1-MSNs and D2-MSNs, predominantly in excitability and latency to first spike.^52^^,^^53^ In line with this, our results indicated higher excitability of D2-MSNs vs. D1-MSNs (Figures S2C and S2D) These inherent differences have concrete implications on information encoding in the two striatal output pathways. *In vivo*, D2-MSNs fire APs at a frequency twice as high as D1-MSNs, which is supported by their low latency.^52^ In agreement with these studies, we found that D2-MSNs exhibited very low latency values during high depolarization (Figure S3F). This low baseline latency explains why MGB activation reduces latency in D1-MSNs, but not in D2-MSNs, thus diminishing the inherent physiological differences between MSN subtypes (Figures 2K and S3F). As coordinated activity of the two striatal output pathways is necessary for complex behaviors, MGB-driven modulation of relative differences between D1-MSNs and D2-MSNs likely has significant implications on network output in TS.^49^^,^^52^

Since our results showed diverse effects of MGB on MSN AP firing, we more closely assessed MSN membrane potential changes in response to optogenetic activation of MGB terminals. We identified several distinct response patterns including excitation/inhibition sequences (Figures 3C–3E, quantified in Table S4). These sequences have previously been reported in anesthetized *in vivo* recordings of cat caudate neurons and rat neostriatal neurons after thalamic stimulation.^54^^,^^55^^,^^56^

Mixed excitatory and inhibitory responses were prominent upon MGB activation with a 20 Hz pulse train (Figures 3D and 3G). Compared to the purely excitatory responses, the mixed responses were characterized by a smaller initial optogenetically evoked excitatory postsynaptic potential (oEPSP) and a persistent reduction in membrane potential that abated only after termination of the light pulses (Figure 3D). Additionally, the average waveform of oEPSPs differed by response type (Figure 3D, insets). oEPSPs from excitation-only responses were broad and did not completely decay before the next pulse, leading to summation and relative depolarization of the membrane potential (Figure 3D, top). In contrast, oEPSPs from mixed responses were narrower and decayed completely or fell below baseline before the next light pulse, thus permitting relative hyperpolarization of the membrane potential (Figure 3D, bottom). This suggests that thalamostriatal afferents induce both transient fluctuations and sustained membrane potential changes in MSNs.

The inhibitory responses observed across stimulation parameters and response types largely exhibited slow dynamics in both current clamp and voltage clamp experiments (Figures 3C–3E and S5A–S5D). The only exception was the excitatory/inhibitory response to the short light pulse, which displayed an initial oEPSP followed by fast hyperpolarization and excitatory rebound (Figure 3C). As GABA_A_ receptors were pharmacologically blocked by picrotoxin, it is unlikely that this hyperpolarization was caused by feedforward inhibition through striatal GABAergic interneurons. However, this postsynaptic potential (PSP) sequence resembles the MSN response dynamics described upon thalamus-induced presynaptic inhibition via M2/4 muscarinic receptors.^11^ In this circuit, thalamostriatal afferents concurrently activate MSNs and striatal cholinergic interneurons. Acetylcholine released from interneurons acts on M2/4 receptors located on excitatory inputs to MSNs. Activation of presynaptic M2/4 receptors transiently suppresses glutamate release onto MSNs, which could explain the fast hyperpolarization we recorded.^11^ In D2-MSNs, this transient inhibition is followed by increased excitability brought on by postsynaptic M1 muscarinic receptors, which tracks with the excitatory rebound we observed.^11^ Therefore, the mixed excitatory/inhibitory response to the short light pulse could be explained by cholinergic signaling in response to thalamostriatal excitation.

Next, we investigated the mechanism underlying slow inhibitory responses observed in the pulse-train and long-pulse conditions (Figures 3D and 3E). GABAergic striatal interneurons produce MSN IPSPs on the millisecond scale and signal through GABA_A_ receptors, which were pharmacologically blocked by picrotoxin throughout all recordings.^34^^,^^35^^,^^57^ We therefore ruled out feedforward mechanisms through striatal interneurons as a source of MGB-TS inhibition.

Instead, we found that MGB-driven hyperpolarization of TS MSNs was monosynaptic and mediated by GABA_B_ receptors (Figures 4A–4E and S6). Slow inhibitory responses were not only significantly reduced by pharmacological blockade of GABA_B_ receptors but also closely resembled recordings of hippocampal and periaqueductal gray neurons after exposure to GABA_B_ receptor agonists such as baclofen.^58^^,^^59^ Moreover, GABA_B_-receptor-mediated signaling explains why slow inhibition was most reliably recorded during long light pulses (500 ms) to activate MGB afferents. Due to their metabotropic nature, GABA_B_-receptor-mediated mechanisms span hundreds, rather than tens, of milliseconds.^58^^,^^59^^,^^60^ Further, GABA_B_ receptors are generally sparse and require pooling of GABA for measurable activation, which, in turn, necessitates potent stimulation of GABAergic neurons.^59^^,^^60^

GABA_B_ receptors can exert inhibitory control through either presynaptic or postsynaptic mechanisms.^60^ In hippocampal neurons, presynaptic GABA_B_ receptors reduce glutamate release by interfering with vesicle fusion.^61^ Postsynaptic GABA_B_ receptors generate slow IPSPs (100–500 ms) by acting through metabotropic pathways with downstream targets including G-protein-coupled inwardly rectifying potassium channels (GIRKs or Kir3) and voltage-gated calcium channels.^62^^,^^63^^,^^64^ Given that TS MSN IPSPs span hundreds of milliseconds and persist in the presence of synaptic blockers for glutamate (Figure S6), we hypothesized that MGB-driven inhibition of TS MSNs is mediated by postsynaptic GABA_B_ receptors. This mechanism would require long-range GABAergic neurons projecting from MGB to TS and would not be entirely without precedent, as several studies have identified inhibitory projections from cortex to striatum, countering the classical idea that corticostriatal projections are solely excitatory.^36^^,^^37^^,^^38^^,^^39^

Indeed, we identified thalamostriatal GABAergic neurons projecting from MGB to TS. These long-range inhibitory neurons were predominantly located in medial MGB (MGBm; Figures 4F–4M and S7). MGBm contains higher-order cells that project to downstream regions including TS and amygdala.^17^^,^^65^ These neurons respond not only to auditory stimuli but also, for example, to visual information, reward, and behavioral context.^17^^,^^65^^,^^66^ To our knowledge, GABAergic neurons have not been previously reported in mouse MGB, though studies have found neurons positive for inhibitory markers in MGB of monkeys and rabbits.^67^^,^^68^

In summary, we show that GABA_B_-receptor-mediated inhibition from MGB extends the dynamic firing range of TS MSNs by delaying depolarization block (see STAR Methods section for definition). The role of slow inhibition in regulating neuronal AP firing has been previously described in the lateral geniculate nucleus, where GABA_B_-receptor-mediated inhibition primes thalamocortical neurons for burst firing.^69^ Therefore, GABA_B_-receptor-mediated inhibition from MGB to TS likely shapes the way in which information is encoded along the thalamostriatal pathway. The inhibitory inputs from MGB to TS are not the only long-range GABAergic neurons in subcortical auditory circuits, as inferior colliculus sends inhibitory afferents to MGB.^70^ Taken together, this points toward an important function for long-range inhibition in thalamostriatal circuits. Our results suggest that long-range inhibitory neurons may be implicated in integrating auditory information for complex behavior and lay the groundwork for investigating this in future *in vivo* studies.

### Limitations of the study

The goal of this study was to examine the modulation of TS output by thalamostriatal afferents from MGB. We demonstrated that MGB neurons provide parallel excitatory and inhibitory GABA_B_-receptor-mediated inputs to TS MSNs that (1) extend high-frequency AP firing during high levels of depolarization and (2) alter temporal coding in a cell type-specific manner. These experiments provide a foundation for future studies to assess MGB-TS projection activity *in vivo* and directly test for neural correlates of learning and behavior.

One important consideration in recording slow MGB-TS inhibition is the length of the light pulse used to activate MGB axon terminals (500 ms). This relatively long stimulation period was required to evoke GABA_B_-receptor-dependent slow hyperpolarization of TS MSNs (Figure 3). Prior studies have noted that GABA_B_ receptor activation requires stronger stimulation parameters to allow for GABA pooling around sparsely distributed GABA_B_ receptors.^60^ While 500 ms light pulses were therefore necessary to observe GABA_B_-receptor-mediated inhibitory responses *ex vivo*, the *in vivo* applicability of this long activation parameter remains to be investigated.

Another key assumption is that cell type classification in TS aligns with other striatal regions. While our knowledge of TS is still limited, it is evident that the cellular architecture is vastly different.^29^ A substantial proportion of MSNs express both D1- and D2-type dopamine receptors, especially in the amygdalostriatal transition zone targeted by MGB projections.^30^ MSNs in TS thus do not segregate cleanly by dopamine receptor subtype, and it is plausible that TS MSN subtypes are defined by entirely different molecular markers.^27^ Here, we use the *Drd1a*-tdTomato mouse line to differentiate between D1-MSNs and D2-MSNs, as this is standard for studies investigating striatal neuron physiology.^71^^,^^72^ However, TS cell types might be more accurately captured by different definitions and molecular profiles in the future.

Finally, we note that all recordings in this study were carried out in the presence of synaptic blockers (picrotoxin) to isolate responses driven by MGB from intrastriatal sources of inhibition. Our results provide valuable insights into the MGB-TS synapse and allowed us to identify a previously unknown source of thalamostriatal inhibition. However, it is also important to consider the circuit in which this synapse is embedded. Previous studies have shown that striatal PV interneurons mediate feedforward inhibition from MGB onto MSNs,^14^^,^^16^ and this should be considered in both the interpretation of our results and the design of future studies.

## Resource availability

### Lead contact

Requests for further information and resources should be directed to the lead contact, Jan Gründemann (jan.grundemann@dzne.de).

### Materials availability

No new materials were generated in this study.

### Data and code availability

• Data: Raw recordings generated in this manuscript have been deposited at Zenodo. The DOI is listed in the key resources table.
• Code: All original code has been deposited at Zenodo and is publicly available. The DOI is listed in the key resources table.
• Additional information: Any additional information is available from the lead contact upon request.

## Acknowledgments

We thank Karl Deisseroth for making AAV2/1-hSyn-hChR2(H134R)-EYFP available. We thank Edward Boyden for making AAVretro-CAG-FLEX-tdTomato available. We thank the DZNE Light Microscopy Facility staff for their technical support. We thank the DZNE Animal Research Facility staff for maintaining the mouse colony, Julia Esser for assistance with genotyping and histology, Eva Sebastian for assistance with injection site coordinates, and Sabine Krabbe and Ricardo Paricio-Montesinos for feedback on the manuscript. This research was supported by the following funding agencies: Boehringer Ingelheim Fonds PhD Fellowship (L.M.H.), 10.13039/501100000781European Research Council (Starting Grant, AXPLAST, J.G.), 10.13039/501100001659Deutsche Forschungsgemeinschaft (SFB1089, SPP2411, J.G.), NRW Forschungsnetzwerk iBehave (J.G.), and the 10.13039/501100005224German Center for Neurodegenerative Diseases (DZNE).

## Author contributions

Conceptualization, L.M.H. and J.G.; formal analysis, L.M.H.; funding acquisition, J.G. and L.M.H.; investigation, L.M.H.; writing – original draft, L.M.H.; methodology, J.G. and L.M.H.; writing – review and editing, J.G. and L.M.H.; visualization, L.M.H.; software, L.M.H.; resources, J.G.; supervision, J.G.

## Declaration of interests

The authors declare no competing interests.

## STAR★Methods

### Key resources table

**Table:** 

REAGENT or RESOURCE	SOURCE	IDENTIFIER
**Antibodies**		

Streptavidin Alexa Fluor^TM^ 647 conjugate	Invitrogen	Cat# S21374; RRID: AB_2336066
Donkey anti-Rabbit IgG (H + L) highly cross-adsorbed Alexa Fluor^TM^ 488	Invitrogen	Cat# A21206; RRID: AB_2535792
Goat anti-calretinin antibody	Swant	Cat# CG1; RRID: AB_10000342
Donkey anti-Goat IgG (H + L) cross-adsorbed Alexa Fluor^TM^ 647	Invitrogen	Cat# A21447; RRID: AB_2535864
Rabbit anti-RFP antibody	Abcam	Cat# ab28664; RRID: AB_777698
Donkey anti-Rabbit IgG (H + L) highly cross-adsorbed Alexa Fluor^TM^ 594	Invitrogen	Cat# A21207; RRID: AB_141637
Guinea pig parvalbumin antibody polyclonal antiserum	Synaptic Systems	Cat# 195 004; RRID: AB_2156476
DyLight^TM^ 405 AffiniPure^TM^ Donkey anti-Guinea Pig IgG (H + L)	Jackson ImmunoResearch	Cat# 706-475-148; RRID: AB_2340470
Mouse GAD67 Monoclonal Antibody	Invitrogen	Cat# MA524909; RRID: AB_2723202
Donkey anti-Mouse IgG (H + L) highly cross-adsorbed secondary antibody, Alexa Fluor^TM^ 488	Invitrogen	Cat# A21202; RRID: AB_141607

**Virus strains**		

AAV2/1-hSyn-hChR2(H134R)-EYFP	Addgene	Cat# 26973-AAV1; RRID: Addgene_26973
AAVretro-CAG-FLEX-tdTomato	Addgene	Cat# 28306-AAVrg; RRID: Addgene_28306

**Chemicals**		

Tetrodotoxin citrate	HelloBio	Cat# HB1035
Picrotoxin	Sigma	Cat# P1675
Biocytin	Sigma	Cat# B4261
ZD-7288	Tocris	Cat# MOLN-M11314
4-aminopyridine	Sigma	Cat# 275875
CNQX	BioTechne	Cat# 1045
D-AP5/APV	HelloBio	Cat# HB0225
CGP 52432	BioTechne	Cat# 1246
QX-314	Sigma	Cat# 552233
Fluoro-Max Color Dyed Blue Aqueous Fluorescent Particles	ThermoScientific	Cat# B500

**Experimental models: Organisms/strains**		

B6.Cg-Tg(Drd1a-tdTomato)6Calak/J transgenic mouseline (*Drd1a*-tdTomato)	Jackson Laboratories	Cat# 016204; RRID: IMSR_JAX:016204
B6J.129S6(FVB)-*Slc32a1*^*tm2(cre)Lowl*^/MwarJ transgenic mouseline (*VGAT*-Cre)	Jackson Laboratories	Cat# 028862; RRID: IMSR_JAX:028862

**Software and algorithms**		

ImageJ	NIH	Link: https://imagej.nih.gov/ij/; RRID: SCR_003070
Axon^TM^ pClamp^TM^ 11 Software Suite	Molecular Devices	Link: https://www.moleculardevices.com/; RRID: SCR_011323
ZEN Microscopy Software version 3.6	Carl Zeiss Microscopy	Link: https://www.zeiss.com; RRID: SCR_013672
Python version 3.12.0	Python.org	Link: https://www.python.org; RRID: SCR_008394
Analysis code	Custom	Link: https://doi.org/10.5281/zenodo.17250452
Data repository	Custom	Link: https://doi.org/10.5281/zenodo.17250420

### Experimental model and study participant details

#### For *ex vivo* animal studies

Female and male mice (5–12 weeks) were used throughout the study. Animals were kept in a 12-h light/dark cycle in ventilated cages with food and water provided *ad libitum*. All procedures were conducted according to institutional guidelines (German Center for Neurodegenerative Diseases (DZNE), Bonn, Germany) and approved by the State Authority for Nature, Environment and Consumer Protection (LANUV NRW, Permit 81–02.04.2023.A043).

### Method details

#### Virus injection for optogenetics

For channelrhodopsin (ChR2) injections, surgeries were performed on *Drd1a-*tdTomato mice (JAX strain #016204) with a stereotaxic apparatus (Kopf Instruments) under isoflurane anesthesia (induction: 5%, maintenance 1–2%). Carprofen (0.067 mg/mL) was administered via the drinking water 24 h prior to and 72 h after the surgery for systemic analgesia. Local analgesia was provided via lidocaine (4 mg/kg) and ropivacaine (2 mg/kg) prior to the incision. 250 nL AAV2/1-hSyn-hChR2(H134R)-EYFP (2.3x10^13^ vg/mL, Addgene) diluted 1:2 in sterile PBS was injected in MGB with a glass pipette, micromanipulator and pressure ejection system (Picospritzer) through a small craniotomy at the following coordinates: AP -3.28, ML -1.98, DV 3.4, 3.3, 3.2. Throughout the entire experimental period, animals were monitored for their wellbeing and given additional fluids and/or analgesia as needed.

#### Acute slice preparation

*Ex vivo* slices were prepared two weeks after the virus injection from animals aged between 55 and 90 days at the time of recording. Slices were collected approximately at the same time of day. Mice were perfused with ice-cold NMDG solution (mM: 2.4 KCl, 1.2 NaH_2_PO_4_, 30 NaHCO_3_, 20 HEPES, 25 Glucose, 3 Na-pyruvate, 5 L-ascorbic acid, 3 myo-inositol, 93 NMDG, 10 MgCl_2_, 0.5 CaCl_2_) bubbled with carbogen gas (98% CO_2_, 2% O_2_) for approximately 30 s, followed by rapid decapitation and removal of the brain. The brain was transferred to ice-cold carbogenated NMDG solution and incubated for 2 min before slices (275 mm) were collected using a Leica VT1200S vibratome. Slices containing MGB were transferred to 4% PFA for fixation and verification of the injection site. Slices containing TS were transferred to 37°C NMDG and incubated for 11–13 min, depending on the age of the animal.^73^ TS slices were then transferred to holding ACSF (mM: 124 NaCl, 2.7 KCl, 26 NaHCO_3_, 1.25 NaH_2_PO_4_, 18.6 glucose, 2.25 L-ascorbic acid, 1.3 MgCl_2_, 2 CaCl_2_) and left to recover for 1 h at room temperature before recording.

#### Electrophysiology

Recordings were performed at room temperature in a slightly modified version of the holding ACSF, which contained elevated CaCl_2_ (3 mM) to enhance release probability from presynaptic terminals. Picrotoxin (50 mM) was added to the recording solution in all experiments to block GABA_A_-receptor-mediated local inhibition. For recordings of monosynaptic activity in current clamp, tetrodotoxin (TTX; 1 mM), ZD-7288 (1 mM) and 4-aminopyridine (4-AP; 0.1 mM) was perfused into the external ACSF.^40^ For GABA_B_-receptor recordings, CGP 52432 (25 mM) was perfused into the monosynaptic current clamp solution. For recordings of monosynaptic activity in voltage clamp, TTX (1 mM), ZD-7288 (1 mM), 4-AP (0.1 mM), CNQX (10 mM) and D-AP5/APV (10 mM) was perfused into the external ACSF. The external solution was constantly bubbled and perfused onto the slices in the recording chamber via a peristatic pump with a flow rate of approximately 2 mL/min.

All recordings were performed using borosilicate pipettes (3–5 MΩ). For current clamp recordings, a potassium-gluconate based internal solution was used, in mM: 130 KGlu, 10 creatine phosphate, 4 MgCl_2_, 3.4 Na_2_ATP, 0.1 Na_3_GTP, 1.1 EGTA and 1 HEPES. The pH was adjusted to 7.4 and the osmolarity was kept at 280–290 mOsm/kg to account for the addition of 2 mg/mL biocytin before recording. For recordings of inhibitory currents in voltage clamp, a caesium-based high chloride internal solution was used, in mM: 110 CsCl, 30 KGlu, 1.1 EGTA, 10 HEPES, 0.1 CaCl_2_, 4 MgATP and 0.3 NaGTP. The pH was adjusted to 7.3 and osmolarity was kept at 280–290 mOsm/kg to account for the addition of 2 mg/mL biocytin and 4 mM QX-314 before recording. For the example recording of an excitatory current in voltage clamp, a CsMeSO_4_-based internal solution was used, in mM: 120 CsCH_3_SO_4_, 5 TEA, 0.4 EGTA, 2.8 MgCl_2_, 2.5 Na_2_ATP, 0.25 Na_3_GTP, 20 HEPES. The pH was adjusted to 7.3 and osmolarity kept at 280–290 mOsm/kg to account for the addition of 2 mg/mL biocytin and 4 mM QX-314 prior to recording.

Cells were visualised through an infrared differential interference contrast-based imaging system using 60× water immersion objective with a numerical aperture of 1.0 (Olympus LUMPLANFL 60x NA 1.0). For recording, MSNs located 3–4 cell layers below the surface of the slice were targeted. MSN subtypes were identified by the presence or absence of td-Tomato from BAC transgenic mice using a fluorescence microscope (Scientifica SliceScope Pro1000, CoolLED pE-300). Cells were filled with biocytin during recording for verification of anatomical location in TS. Biocytin filling was also used to confirm MSN-like morphology (radial dendrites with dense spines) as to discard recordings from tdTomato-negative interneurons (sparse and aspiny dendrites). Data were collected using a Multiclamp 700B amplifier (Axon Instruments), Axon Digidata 1550B digitiser (Molecular Devices), and pClamp 11 recording software (Molecular Devices). Signals were sampled at 50 kHz and analyzed in Python.

ChR2 was activated using a blue light-emitting diode (470 nm) and driver (CoolLED pE-300 Ultra) through a 60× objective with a power range of 0.029–0.282 mW/mm^2^. LED intensity was adjusted for each cell to the minimum power required to elicit a postsynaptic potential. In current clamp, postsynaptic potentials were recorded during 10 ms and 500 ms single pulses, and pulse trains consisting of 5 ms pulses at 1 Hz, 5 Hz, 10 Hz and 20 Hz. In voltage clamp, postsynaptic currents were recorded during 10 ms and 500 ms single pulses, and 20 Hz pulse trains consisting of 5 ms. For monosynaptic activity voltage-clamp recordings, postsynaptic currents were recorded during 2 ms and 10 ms single pulses, as well as 20 Hz pulse trains consisting of 2 ms and 10 ms pulses. Action potential activity was recorded in current clamp by applying successively increasing depolarising current steps at 50 pA increments until the cell reached depolarization block. For each current step, a corresponding spike train was recorded 5 s later simultaneously with 500 ms light pulse. Current clamp recordings were performed with bridge balance compensation. Cells were held at 0 pA during all current clamp recordings, except for CGP 52432 wash-on experiments, where holding current was injected to match pre-wash-on membrane potential to avoid confounds of electrical driving force on current magnitude.

Access resistance was readout online from Clampex and confirmed with a −10 mV test pulse acquired voltage clamp mode before and after current clamp recordings. During voltage-clamp recordings, cells were held at −70 mV. R_a_ was not compensated and cells with R_a_ larger than 30 MΩ were discarded. Cells that exhibited fast-spiking properties and morphologies characteristic of interneurons were excluded from the analysis. Cells that failed to produce continuous spike trains were also discarded. Recordings where postsynaptic potentials could not be reliably categorised due to noise or fluctuations in membrane potential were also discarded. Analysis and plotting of electrophysiology data was performed via custom-made Python scripts using the pyABF library.

#### Histology

After recording, slices were fixed in 4% PFA for 24 h before being transferred to 1x PBS. Slices were washed 3 times for 20 min each in 1x PBS and then blocked for 2 h at room temperature with solution containing 10% normal horse serum and 0.5% Triton X- in PBS. Slices containing MGB were incubated with a primary antibody against calretinin (1:1000, Swant). Slices containing TS were incubated with a primary antibody against RFP (1:1000, Abcam). Both MGB and TS slices were incubated with their respective primary antibodies overnight at 4°C, then washed 3 times for 20 min each in 1x PBS containing 0.1% Triton X-. Subsequently, slices containing MGB were incubated with an anti-goat 647 secondary antibody (1:1000, Invitrogen) and slices containing TS were incubated with a streptavidin-647 direct antibody (1:1000, Invitrogen) and an anti-rabbit 594 secondary antibody (1:1000, Invitrogen) to boost intrinsic tdTomato signal. Slices were incubated with their respective secondary antibodies for 2 h at room temperature. Finally, slices were washed 3 times for 20 min each in PBS and mounted onto Superfrost microscope slides. To check the specificity of the injection site, location and morphology of recorded cells, stained slices were imaged on a Zeiss LSM900 confocal microscope through 2.5x, 10x and 20x objectives.

#### Anatomical tracing

For retrograde tracing of long-range inhibitory projections, conditionally expressed AAVretro-CAG-FLEX-tdTomato (2.6x10^13^ vg/mL, Addgene) was unilaterally injected into tail striatum (AP -1.3, ML -3.4, DV 3.35, 3.25, 3.15) of *VGAT*-Cre mice. The injection was supplemented with non-retrograde blue dyed microspheres (1:1400, ThermoScientific) to provide accurate indication of the injection site.

Two weeks after the injection, mice were perfused with 1x PBS followed by 4% PFA. Brains were kept in PFA for 24 h before transfer to 1x PBS until slicing. 50 mm slices of both MGB and TS were made using a Leica VT1000S vibratome. Blocking, washing and incubation steps were identical to the protocol described above. MGB slices from retrograde tracing injections were counterstained with primary antibodies against RFP (1:1000, Abcam), PV (1:500, Synaptic Systems), GAD67 (1:50, Invitrogen) and calretinin (1:1000, Swant). TS slices from retrograde tracing injections were counterstained against RFP (1:1000, Abcam). MGB slices were then incubated in an anti-rabbit 594 secondary antibody (1:1000, Invitrogen), anti-guinea pig 405 secondary antibody (1:400, Jackson ImmunoResearch), anti-mouse 488 secondary antibody (1:1000, Invitrogen) and an anti-goat 647 secondary antibody (1:1000, Invitrogen). TS slices were incubated in an anti-rabbit 594 secondary antibody (1:1000, Invitrogen). 11 slices from 2 mice were imaged with a Zeiss LSM900 confocal microscope through 10x and 20x objectives.

### Quantification and statistical analysis

All details for statistical comparisons are listed in Table S1. Where statistical tests are presented as asterisks, these refer to Wilcoxon signed-rank tests with the following *p*-values: *p* < 0.05 (∗), *p* < 0.01(∗∗), *p* < 0.001(∗∗∗), *p* < 0.0001(∗∗∗∗). The recorded MSNs came from 18 mice. Details on the number of slices and mice used for each set of recordings is provided in the figure legends. All data was extracted, analyzed and plotted in custom-made Python (Version 3.12.0) scripts using the pyABF library (https://pypi.org/project/pyabf/). Statistical analyses were performed using SciPy statistics (https://docs.scipy.org/doc/scipy/index.html), statsmodels (https://www.statsmodels.org/stable/index.html) and scikit-posthoc (https://pypi.org/project/scikit-posthocs/) modules. A two-way ANOVA with an ordinary least squares (OLS) regression model was used to compare I/O curves. For comparisons between paired data points (light vs. no light, pre-vs. post-CGP), a Wilcoxon signed-rank test was applied, with a Bonferroni correction for multiple comparisons as needed. For comparisons between unpaired data points (D1-MSNs vs. D2-MSNs), a Mann-WhitneyU post-hoc test was used. For comparisons of response distributions across stimulation parameters, a chi-squared test was used.

For measurement of action potential (AP) properties, values categorised under “first spike” refer to the first AP in the spike train. Values categorised under “spike train mean” refer to the average of all APs in the train, excluding the first AP. ISI was always calculated as an average from all APs in the train. To calculate latency to first AP, the first sweep at which APs were observed in both without and with optogenetic activation conditions was used. ISI for the low depolarisation condition was computed one sweep above the first APs, as to allow for quantification of intervals between multiple APs. To precisely determine the sweep at which the cell entered depolarisation block, a histogram with all AP heights in the spike train was computed. This gave a bimodal distribution, with the higher histogram peak representing the height of full APs and the lower histogram peak representing the height of attenuated APs. The first sweep at which half of the APs in the spike train fell below the lower histogram peak was defined as depolarisation block. This sweep was used for all measurements in the high depolarisation condition. For all AP property measurements, including firing frequency, measurements from spike trains without and with optogenetic stimulation came from the same sweep and were therefore subject to paired statistical comparisons.
