# Supplementary material for: Inhibitory inputs from thalamus promote resilient spiking in tail of striatum
Source: iScience. 2025 Oct 27;28(11):113880. doi: 10.1016/j.isci.2025.113880 (PMC12682267; doi:10.1016/j.isci.2025.113880)
Supplement: Document S1. Figures S1–S7 and Tables S1–S4 [file mmc1.pdf]

iScience, Volume *28*

Supplemental information

Inhibitory inputs from thalamus promote resilient spiking in tail of striatum

Laura M. Haetzel and Jan Gründemann

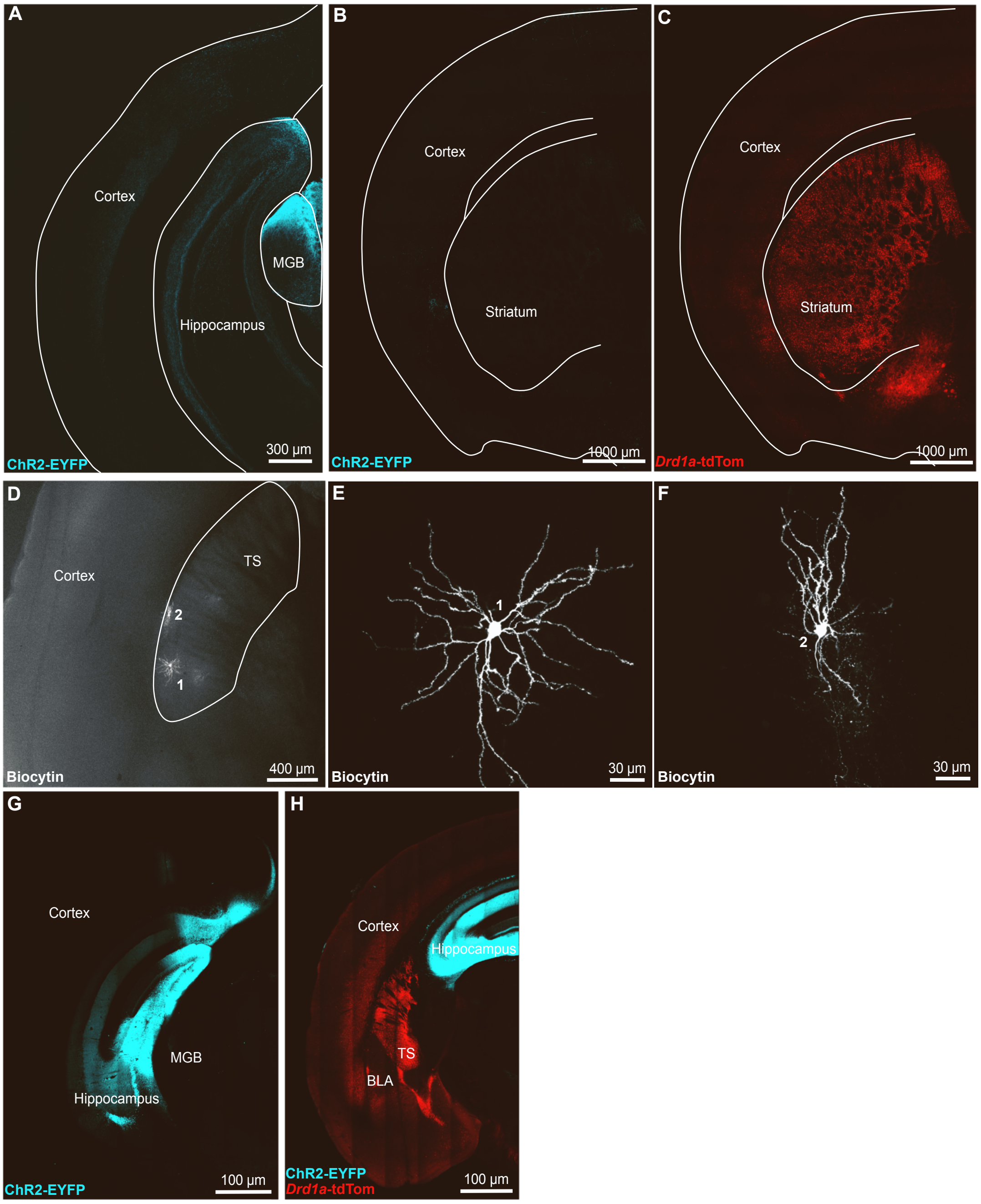

Supplemental Figure 1: Projections surrounding MGB and TS (related to Figure 1). (A) Injection of ChR2 into MGB shows (B) no fibers terminating in (C) anterior striatum. (D) Example slice fixed after recording showing (E-F) two biocytin-filled cells in TS. (G) Injection of ChR2 in hippocampal area surrounding MGB (H) does not indicate extrahippocampal projections in TS or other areas within the coronal plane. Scale bar lengths A: 300 μm, B-C: 1000 μm, D: 400 μm, E-F: 30 μm, G-H: 100 μm.

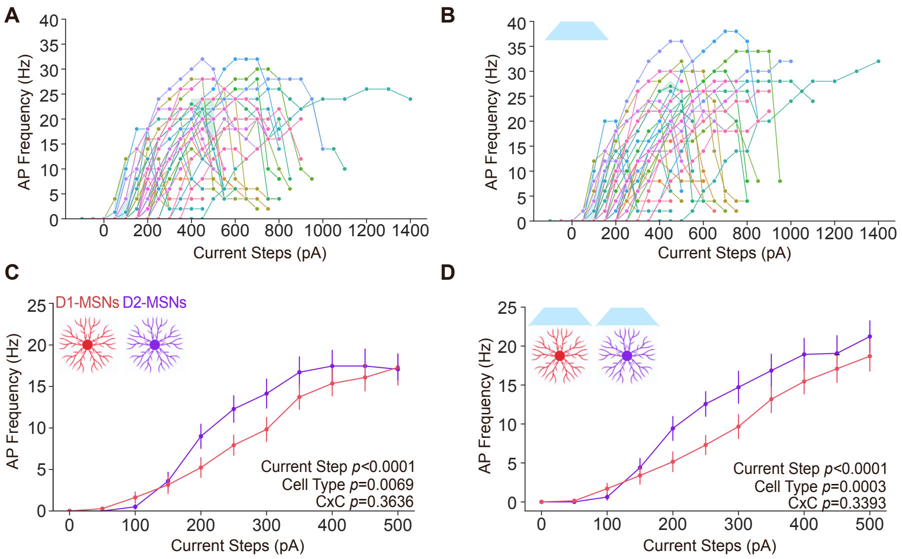

Supplemental Figure 2: D2-MSNS exhibit higher intrinsic excitability than D1-MSNs (related to Figure 2). (A) individual F/I curves for all recorded cells with AP Frequency plotted against current step size without and (B) with optogenetic stimulation. (C) Average F/I curves for D1-MSNs (red, mean±SEM) and D2-MSNs (purple, mean±SEM) for current steps ranging from 0 to 500 pA without and (D) with optogenetic stimulation (mean±SEM, *n*=31 D1-MSNs, 14 D2-MSNs, from 20 slices across 8 mice, Two-way ANOVA) .

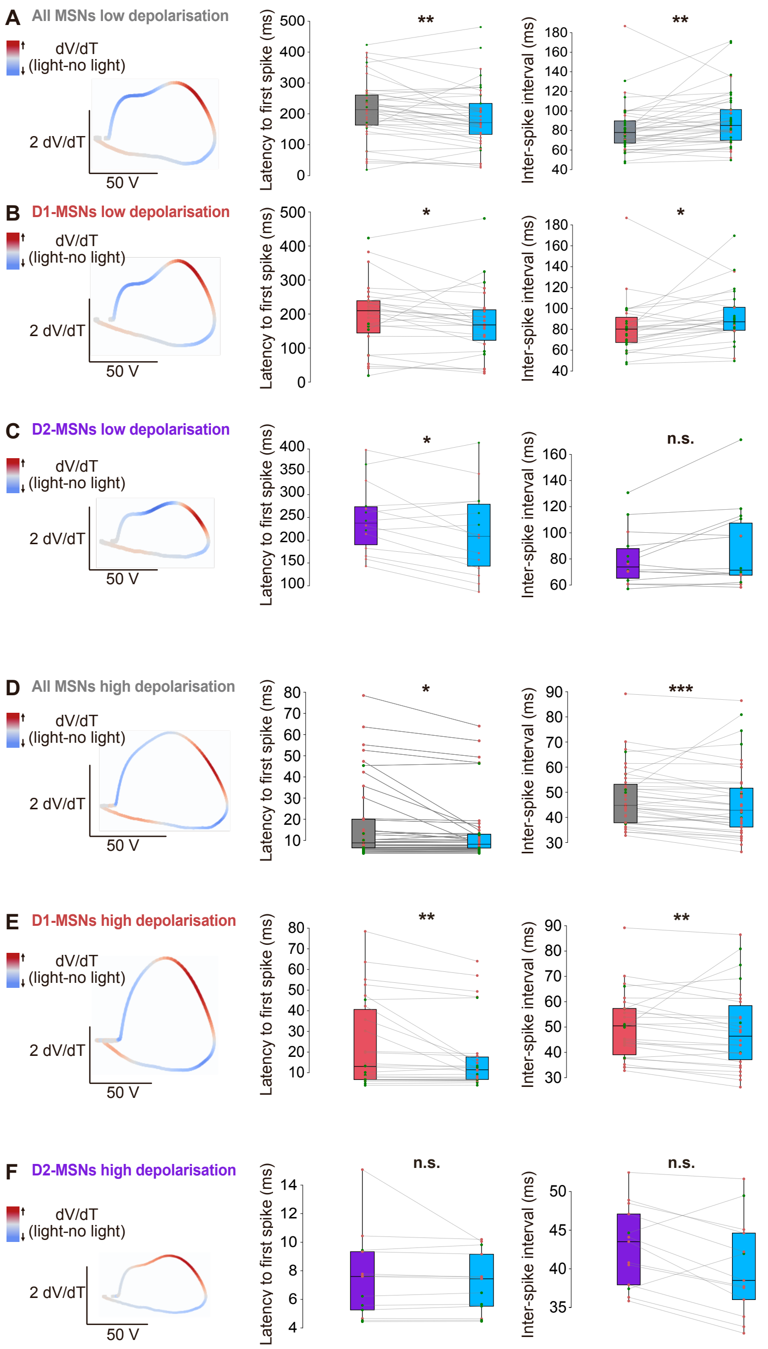

Supplemental Figure 3: MGB broadly alters MSN action potential dynamics (related to Figure 2). (A) Left: Phase plot with heat map (light-no light) showing changes in action potential dynamics of all MSNs during early current steps after optogenetic activation of MGB (represented as mean heat map from 41 cells superimposed on a phase plot from one example cell). Right: Breakdown of latency and ISI differences for all MSNs in Figures 2H and 2J (represented as mean with interquartile range; Wilcoxon signed-rank test; **: $p<0.01$). (B) Left: Phase plot with heat map (light-no light) showing changes in D1-MSN action potential dynamics during early current steps (represented as mean heat map from 27 cells superimposed on a phase plot from one example cell). Right: Breakdown of latency and ISI changes for D1-MSNs in 2H and 2J (represented as mean with interquartile range; Wilcoxon signed-rank test; *: $p<0.05$). (C) Left: Phase plot with heat map (light-no light) showing changes in D2-MSN action potential dynamics during early current steps (represented as mean heat map from 14 cells superimposed on a phase plot from one example cell). Right: Breakdown of latency and ISI changes for D2-MSNs in 2H and 2J (represented as mean with interquartile range; Wilcoxon signed-rank test; *: $p<0.05$). (D) Left: Phase plot with heat map (light-no light) showing changes in MSN action potential dynamics during late current steps (represented as mean heat map from 37 cells superimposed on a phase plot from one example cell). Right: Breakdown of latency and ISI differences for all MSNs in 2I and 2K (represented as mean with interquartile range; Wilcoxon signed-rank test; *: $p<0.05$, ***: $p<0.001$). (E) Left: Phase plot with heat map (light-no light) showing changes in D1-MSN action potential dynamics during late current steps (represented as mean heat map from 24 cells superimposed on a phase plot from one example cell). Right: Breakdown of latency and ISI differences for D1-MSNs in 2I and 2K (represented as mean with interquartile range; Wilcoxon signed-rank test; **: $p<0.01$). (F) Left: Phase plot with heat map (light-no light) showing changes in D2-MSN action potential dynamics during late current steps (represented as mean heat map from 13 cells superimposed on a phase plot from one example cell). Right: Breakdown of latency and ISI differences for D2-MSNs in 2I and 2K (represented as mean with interquartile range; Wilcoxon signed-rank test).

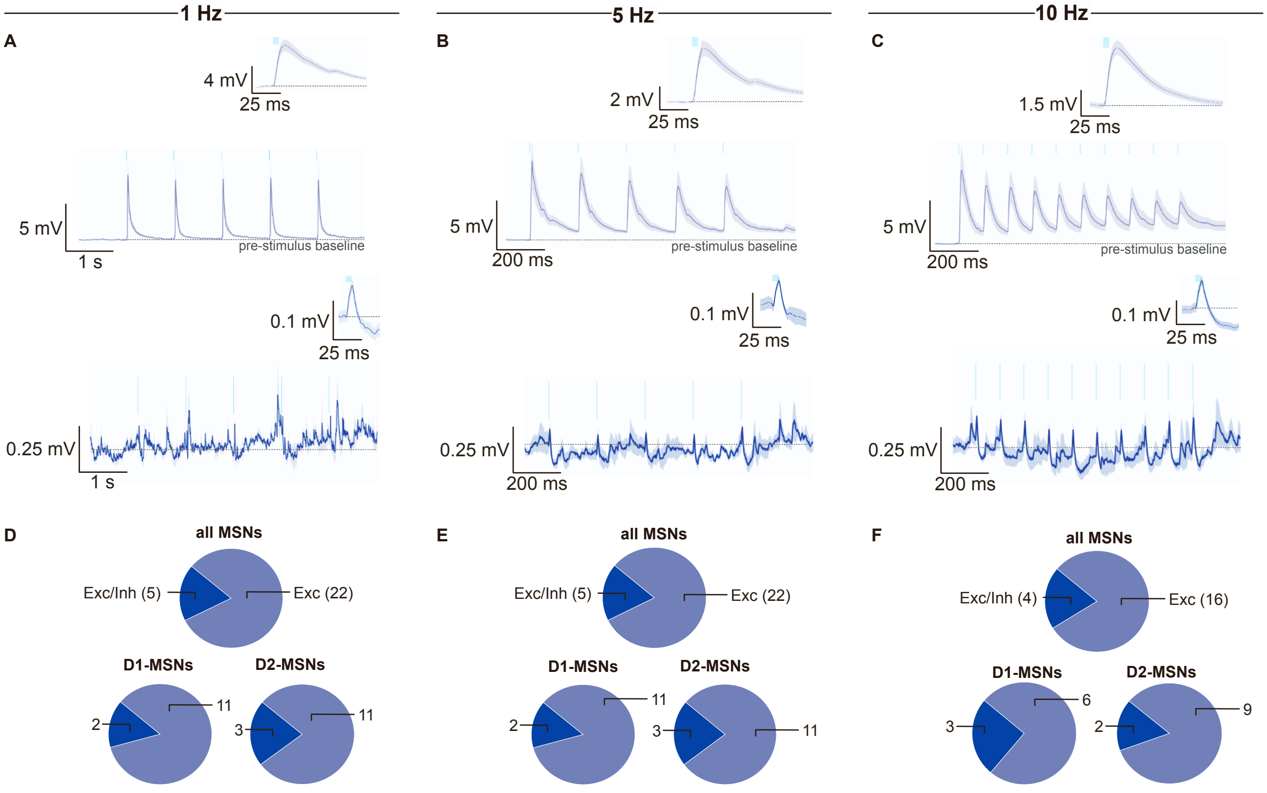

Supplemental Figure 4: Additional characterisation of TS responses to MGB activation at different frequencies (related to Figure 3). (A) Average trace of all excitatory responses (light blue, mean of 22 cells±SEM) and mixed excitatory/inhibitory responses (blue, mean of 5 cells±SEM) in response to a 1 Hz pulse train lasting for 5 seconds. (B) Average trace of all excitatory responses (light blue, mean of 22 cells±SEM) and mixed excitatory/inhibitory responses (blue, mean of 5 cells±SEM) in response to a 5 Hz pulse train lasting for 1 second. (C) Average trace of all excitatory responses (light blue, mean of 16 cells±SEM) and mixed excitatory/inhibitory responses (blue, mean of 4 cells±SEM) in response to a 10 Hz pulse train lasting for 1 second. (D) Breakdown of response types to 1 Hz stimulation for all MSNs (*n*=27 cells from 11 slices across 5 mice; 81% excitation, 19% mixed), D1-MSNs (*n*=13 cells from 11 slices across 5 mice; 85% excitation, 15% mixed) and D2-MSNs (*n*=14 cells from 10 slices across 5 mice, 79% excitation, 21% mixed). (E) Breakdown of response types to 5 Hz stimulation for all MSNs (*n*=27 cells from 11 slices across 5 mice; 81% excitation, 19% mixed), D1=MSNs (*n*=13 cells from 11 slices across 5 mice; 85% excitation, 15% mixed) and D2-MSNs (*n*=14 cells from 10 slices across 5 mice, 79% excitation, 21% mixed). (F) Breakdown of response types to 10 Hz stimulation for all MSNs (*n*=20 cells from 7 slices across 3 mice; 80% excitation, 20% mixed), D1-MSNs (*n*=9 cells from 6 slices across 3 mice; 67% excitation, 33% mixed) and D2-MSNs (*n*=11 cells from 7 slices across 3 mice, 82% excitation, 18% mixed).

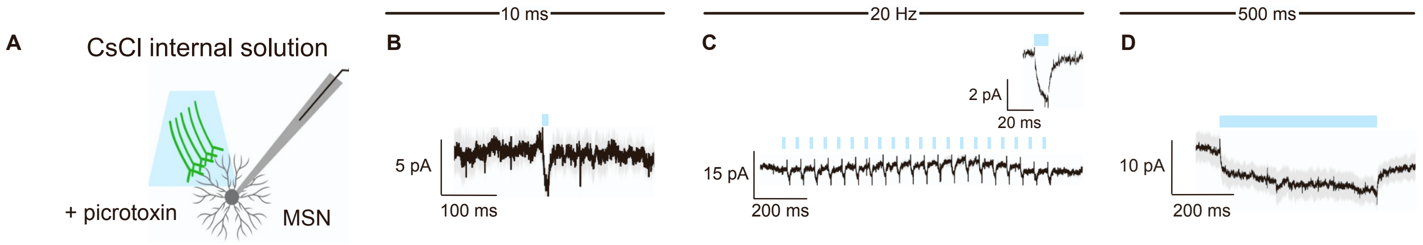

Supplemental Figure 5: Inhibitory currents in response to different stimulation protocols characterised in voltage clamp (related to Figure 3). (A) Recording setup with high-chloride internal to highlight inhibitory currents and picrotoxin to block local microcircuit activity (B) Responses to 10 ms, (C) 20 Hz and (D) 500 ms light pulses recorded in voltage clamp with CsCl internal solution, clamped at -70 mV in the presence of picrotoxin (mean of *n*=9 cells±SEM from 4 slices across 3 mice, 5 D1-MSNs and 4 D2-MSNs).

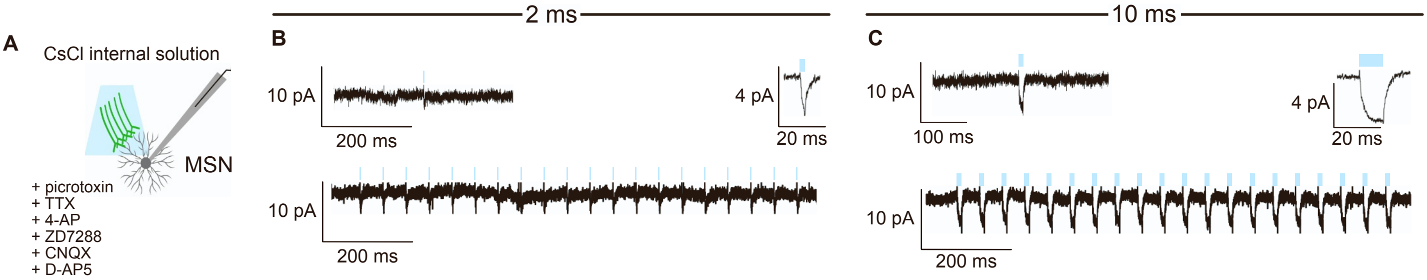

Supplemental Figure 6: Inhibitory monosynaptic MGB-TS inputs recorded in voltage clamp (related to Figure 4). (A) Recording protocol with CsCl internal solution and synaptic blockers to target slow monosynaptic inhibition (B) Response to a single 2 ms light pulse (mean of 9 cells±SEM, top left), a 20Hz pulse train consisting of 2ms pulses (mean of 9 cells±SEM, bottom) and the average of all pulses (mean±SEM, top right inset) (C) Response to a single 10 ms light pulse (top left), a 20Hz pulse train consisting of 10 ms pulses (mean of 9 cells±SEM, bottom) and the average of all pulses (mean±SEM, top right inset) (n=9 cells from 3 slices taken from 2 mice. Recordings of 2 ms and 10 ms light pulses were conducted in the same cells, light artefacts cropped out of recordings).

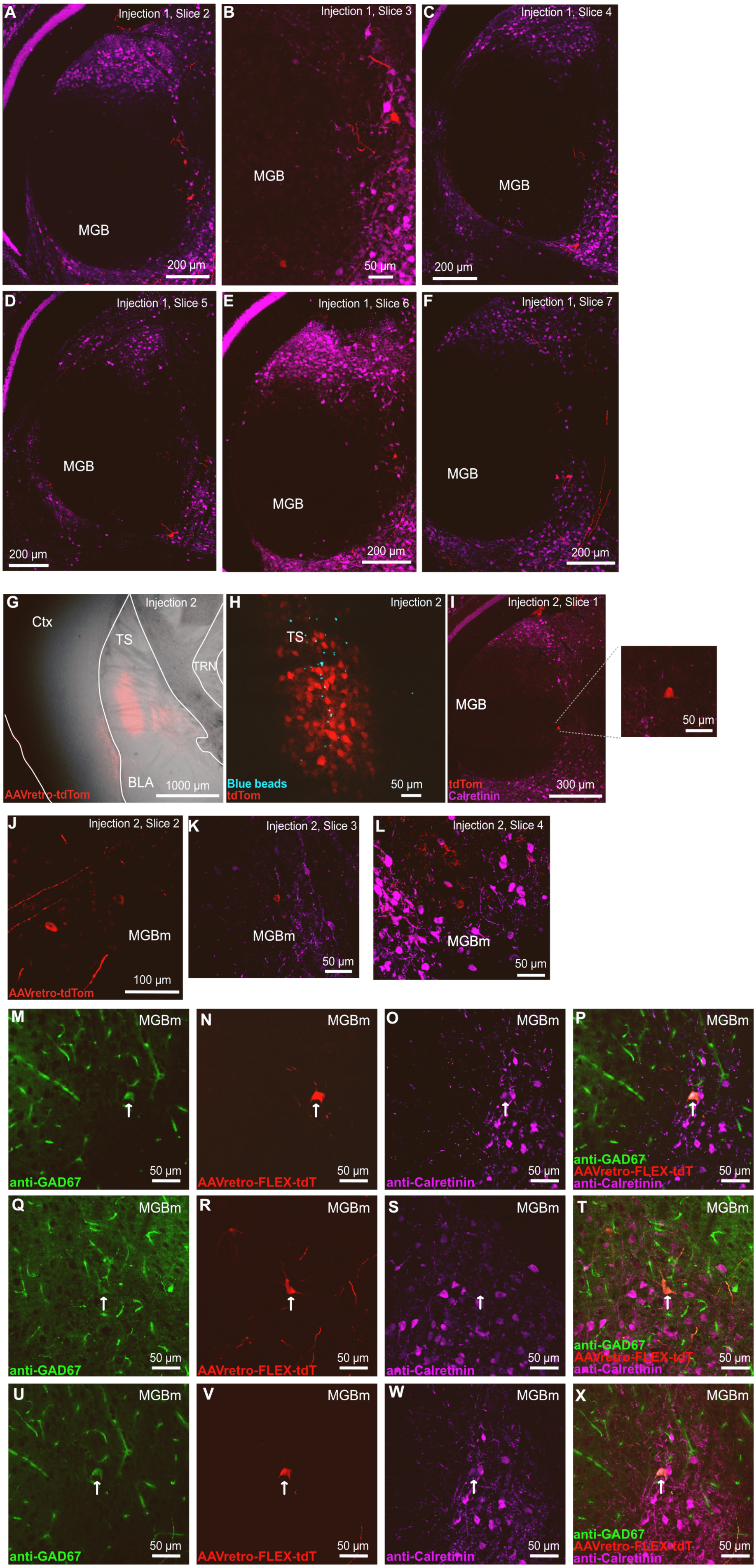

Supplemental Figure 7: Biological replicates for identification of long-range GABAergic neurons projecting from MGB to TS (related to Figure 4). (A-L) Biological replicates for the retrograde tracing experiment shown in Figure 4F. Conditionally labelled cell bodies in MGB of *VGAT*-Cre mice were replicated in 11 slices across 2 mice. Scale bar length A: 200 µm, B: 50 µm, C-F: 200 µm, G: 1000 µm, H: 50 µm, I: 300 µm, I (inset): 50 µm, J: 100 µm, K-L: 50 µm. (M-X) Biological replicates for Figures 4J-M, showing 3 additional MGB neurons co-expressing GAD67 and conditionally expressed AAVretro-FLEX-tdTomato following injection in TS of *VGAT*-Cre mice. Co-expressing cells were replicated in 4 slices across 2 mice. Scale bar length M-X: 50 µm.

Supplemental Table 1: Summary of statistical tests for all figures

Figure	Experiment	Statistical test	n	Statistical test values
**2F**	All MSNs I/O curve (low depolarisation)	Two-way ANOVA	41	LED: *p*=0.450, F=0.572<br>Current: *p*<0.0001, F=98.36 LEDxCurrent: *p*=0.984, F=0.051
**2F (top inset)**	D1-MSNs I/O curve (low depolarisation)	Two-way ANOVA	27	LED: *p*=0.2502, F=1.33<br>Current: *p*<0.0001, F=64.41 LEDxCurrent: *p*=0.987, F=0.08
**2F (bottom inset)**	D2-MSNs I/O curve (low depolarisation)	Two-way ANOVA	14	LED: *p*=0.7006, F=0.15<br>Current: *p*<0.0001, F=27.44 LEDxCurrent: *p*=0.9994, F=0.02
**2G**	All MSNs I/O curve (high depolarisation)	Two-way ANOVA	36	LED: *p*=0.0004, F=12.83<br>Current: *p*<0.0001, F=8.12<br>LEDxCurrent: *p*=0.565, F=0.81
**2G**	All MSNs I/O curve (high depolarisation)	Wilcoxon signed-rank test with Bonferroni correction	36	-300 pA: *p*=0.615<br>-250 pA: *p*=0.1914, W-statistic=156.5<br>-200 pA: *p*=0.0032, W-statistic=87.5<br>-150 pA: *p*=0.0025, W-statistic=57.5<br>-100 pA: *p*<0.0001, W-statistic=13.0<br>-50 pA: *p*<0.0001, W-statistic=24.0<br>0 pA: *p*=0.0030, W-statistic=47.0
**2G (top inset)**	D1-MSNs I/O curve (high depolarisation)	Two-way ANOVA	23	LED: *p*=0.0101, F=6.71<br>Current: *p*=0.0003, F=4.41<br>LEDxCurrent: *p*=0.608, F=0.61
**2G (top inset)**	D1-MSNs I/O curve (high depolarisation)	Wilcoxon signed-rank test with Bonferroni correction	23	-300 pA: *p*=0.7963, W-statistic=56.0<br>-250 pA: *p*=0.5272, W-statistic=43.0<br>-200 pA: *p*=0.0046, W-statistic=3.5<br>-150 pA: *p*=0.0018, W-statistic=15.0<br>-100 pA: *p*=0.0009, W-statistic=0<br>-50pA: *p*=0.0003, W-statistic=2.5<br>0 pA: *p*=0.0155, W-statistic=11.0
**2G (bottom inset)**	D2-MSNs I/O curve (high depolarisation)	Two-way ANOVA	13	LED: *p*=0.0137, F=6.21<br>Current: *p*=0.0002, F=4.65<br>LEDxCurrent: *p*=0.9667, F=0.23
**2G (bottom inset)**	D2-MSNs I/O curve (high depolarisation)	Wilcoxon signed-rank test with Bonferroni correction	13	-300 pA *p*=0.2482, W-statistic=13.5<br>-250 pA *p*=0.0349, W-statistic=4.0<br>-200 pA *p*=0.0066, W-statistic=0.0<br>-150 pA *p*=0.0164, W-statistic=2.5<br>-100 pA *p*=0.0071, W-statistic=0.0<br>-50 pA *p*=0.0176, W-statistic=0.0<br>0 pA *p*=0.0650, W-statistic=5.0
**S2C**	D1-MSNs vs. D2-MSNs F/I curve without optogenetics	Two-way ANOVA	0 pA: 40<br>50 pA: 43<br>100 pA: 43<br>150 pA: 41<br>200 pA: 40<br>250 pA: 40<br>300 pA: 38<br>350 pA: 36<br>400 pA: 37<br>450 pA: 37<br>500 pA: 33	Current *p*<0.0001, F=54.27<br>Cell Type *p*=0.0069, F=7.36<br>CurrentxCell Type *p*=0.3636, F=1.10
**S2C**	D1-MSNs vs. D2-MSNs F/I curve without optogenetics	Mann-WhitneyU	(see above)	0 pA: *p*=1.0<br>50 pA: *p*=0.3112<br>100 pA: *p*=0.5188<br>150 pA: *p*=0.3635<br>200 pA: *p*=0.3280<br>250 pA: *p*=0.3412<br>300 pA: *p*=0.6113<br>350 pA: *p*=0.1463<br>400 pA: *p*=0.2487<br>450 pA: *p*=0.5849<br>500 pA: *p*=0.9852
**S2D**	D1-MSNs vs. D2-MSNs F/I curve with optogenetics	Two-way ANOVA	(see above)	Current *p*>0.0001, F=51.84<br>Cell Type *p*=0.0003, F=13.20<br>CurrentxCell Type *p*=0.3394, F=1.13
**S2D**	D1-MSNs vs. D2-MSNs F/I curve with optogenetics	Mann-WhitneyU	(see above)	0 pA: *p*=1.0<br>50 pA: *p*=0.3112<br>100 pA: *p*=0.5063<br>150 pA: *p*=0.2198<br>200 pA: *p*=0.2787<br>250 pA: *p*=0.1309<br>300 pA: *p*=0.7226<br>350 pA: *p*=0.1753<br>400 pA: *p*=0.1058<br>450 pA: *p*=0.2978<br>500 pA: *p*=0.3093
**2H (left) and S3A (left)**	All MSNs latency change (low depolarisation)	Wilcoxon signed-rank test	41	*p*=0.005, W-statistic=218.0.
**2H (middle) and S3B (left)**	D1-MSNs latency change (low depolarisation)	Wilcoxon signed-rank test	27	*p*=0.046, W-statistic=106.0
**2H (right) and S3C (left)**	D2-MSNs latency change (low depolarisation)	Wilcoxon signed-rank test	14	*p*=0.0295, W-statistic=18.0
**2I (left) and S3D (left)**	All MSNs latency change (high depolarisation)	Wilcoxon signed-rank test	37	*p*=0.012, W-statistic=212.0
**2I (middle) and S3E (left)**	D1-MSNs latency change (high depolarisation)	Wilcoxon signed-rank test	24	*p*=0.0086, W-statistic=74.0
**2I (right) and S3F (left)**	D2-MSNs latency change (low depolarisation)	Wilcoxon signed-rank test	13	*p*=0.7869, W-statistic=40.5
**2J (left) and S3A (right)**	All MSNs ISI change (low depolarisation)	Wilcoxon signed-rank test	41	*p*=0.002, W-statistic=197.0
**2J (middle) and S3B (right)**	D1-MSNs ISI change (low depolarisation)	Wilcoxon signed-rank test	27	*p*=0.0121, W-statistic=86.0

**2J (right) and S3C (right)**	D2-MSNs ISI change (low depolarisation)	Wilcoxon signed-rank test	14	p=0.1726, W-statistic=30.0
**2K (left) and S3D (right)**	All MSNs ISI change (high depolarisation)	Wilcoxon signed-rank test	37	p=0.0006, W-statistic=151.0
**2K (middle) and S3E (right)**	D1-MSNs ISI change (high depolarisation)	Wilcoxon signed-rank test	24	p=0.0079, W-statistic=73.0
**2K (right) and S3F (right)**	D2-MSNs ISI change (high depolarisation)	Wilcoxon signed-rank test	13	p=0.0681, W-statistic=19.0
**3F, 3G, 3H**	All MSNs response distributions	χ^2 test	57	10 ms vs. 500 ms: p<0.0001<br>10 ms vs. 20 Hz: p=0.1289<br>20 Hz vs. 500 ms: p<0.0001
**3F, 3G, 3H**	D1-MSNs response distributions	χ^2 test	34	10 ms vs. 500 ms: p<0.0001<br>10 ms vs. 20 Hz: p=0.3664<br>20 Hz vs. 500 ms: p=0.0003
**3F, 3G, 3H**	D2-MSNs response distributions	χ^2 test	23	10 ms vs. 500 ms: p<0.0001<br>10 ms vs. 20 Hz: p=0.2622<br>20 Hz vs. 500 ms: p=0.0002
**4E**	Vm change pre vs. post CGP wash-on	Wilcoxon signed-rank test	9	p=0.0019
**Supplemental Table 2**	All MSNs AP properties early depolarisation (light vs. no light)	Wilcoxon signed-rank test	41	Threshold first spike: p=0.0229, W-statistic=301.0<br>Threshold mean of spikes: p=0.0021, W-statistic=224.0<br>Height first spike: p=0.0294, W-statistic=309.0<br>Height mean of spikes: p=0.2418, W-statistic=375.0<br>AHP first spike: p=0.0005, W-statistic=192.0<br>AHP mean of spikes: p=0.0669, W-statistic=103.0<br>Width first spike: p<0.0001, W-statistic=44.0<br>Width mean of spikes: p>0.0001, W-statistic=0<br>Rise time first spike: p<0.0001, W-statistic=0<br>Rise time mean of spikes: p<0.0001, W-statistic=24.0<br>ISI mean of spikes: p=0.002, W-statistic=197.0
**Supplemental Table 2**	D1-MSNs AP properties early depolarisation (light vs. no light)	Wilcoxon signed-rank test	27	Threshold first spike: p=0.229, W-statistic=161.0<br>Threshold mean of spikes: p=0.0089, W-statistic=90.0<br>Height first spike: p=0.0365, W-statistic=121.0<br>Height mean of spikes: p=0.9553, W-statistic=200.0<br>AHP first spike: p=0.0041, W-statistic=80.0<br>AHP mean of spikes: p=0.0084, W-statistic=15.0<br>Width first spike: p<0.0001, W-statistic=0.0<br>Width mean of spikes: p<0.0001, W-statistic=0.0<br>Rise time first spike: p<0.0001, W-statistic=0.0<br>Rise time mean of spikes: p<0.0001, W-statistic=12.0<br>Latency first spike: p=0.0463, W-statistic=106.0<br>ISI mean of spikes: p=0.0121, W-statistic=86.0
**Supplemental Table 2**	D2-MSNs AP properties early depolarisation (light vs. no light)	Wilcoxon signed-rank test	14	Threshold first spike: p=0.0256, W-statistic=21.0<br>Threshold mean of spikes: p=0.2769, W-statistic=40.0<br>Height first spike: p=0.5995, W-statistic=50.0<br>Height mean of spikes: p=0.0103, W-statistic=16.0<br>AHP first spike: p=0.1040, W-statistic=26.0<br>AHP mean of spikes: p=0.7695, W-statistic=24.0<br>Width first spike: p=0.0084, W-statistic=15.0<br>Width mean of spikes: p=0.0002, W-statistic=0<br>Rise time first spike: p<0.0001, W-statistic=0.0<br>Rise time mean of spikes: p=0.0084, W-statistic=15.0<br>Latency first spike: p=0.0295, W-statistic=18.0<br>ISI mean spikes: p=0.1726, W-statistic=30.0
**Supplemental Table 3**	All MSNs AP properties late depolarisation (light vs. no light)	Wilcoxon signed-rank test	37	Threshold first spike: p=0.0420, W-statistic=289.0<br>Threshold mean of spikes: p=0.0002, W-statistic=168.0<br>Height first spike: p=0.5688, W-statistic=405.0<br>Height mean of spikes: p<0.0001, W-statistic=57.0<br>AHP first spike: p=0.0002, W-statistic=168.0<br>AHP mean of spikes: p=0.5522, W-statistic=403.0<br>Width first spike: p<0.0001, W-statistic=41.5<br>Width mean of spikes: p<0.0001, W-statistic=0.0<br>Rise-time first spike: p=0.0020, W-statistic=109.0<br>Rise-time mean of spikes: p<0.0001, W-statistic=9.5<br>Latency first spike: p=0.0121, W-statistic=212.0<br>ISI mean of spikes: p=0.0006, W-statistic=151.0
**Supplemental Table 3**	D1-MSNs AP properties late depolarisation (light vs. no light)	Wilcoxon signed-rank test	24	Threshold first spike: p=0.2359, W-statistic=150.0<br>Threshold mean of spikes: p=0.0095, W-statistic=91.0<br>Height first spike: p=0.2359, W-statistic=150.0<br>Height mean of spikes: p<0.0001, W-statistic=35.0<br>AHP first spike: p=0.0095, W-statistic=91.0<br>AHP mean of spikes: p=0.9375. W-statistic=199.0<br>Width first spike: p<0.0001, W-statistic=7.0<br>Width mean of spikes: p<0.0001, W-statistic=0.0<br>Rise time first spike: p=0.0021, W-statistic=37.5<br>Rise time mean of spikes: p<0.0001, W-statistic=8.5<br>Latency first spike: p=0.7867, W-statistic=40.5<br>ISI mean of spikes: p=0.0079, W-statistic=73.0
**Supplemental Table 3**	D2-MSNs AP properties late depolarisation (light vs. no light)	Wilcoxon signed-rank test	13	Threshold first spike: p=0.1677, W-statistic=25.0<br>Threshold mean of spikes: p=0.013, W-statistic=11.0<br>Height first spike: p=0.6355, W-statistic=38.0<br>Height mean of spikes: p=0.0004, W-statistic=1.0<br>AHP first spike: p=0.0266, W-statistic=14.0<br>AHP mean of spikes: p=0.3396, W-statistic=31.0<br>Width first spike: p=0.0546, W-statistic=11.5<br>Width mean of spikes: p=0.0002, W-statistic=0.0<br>Rise time first spike: p=0.4331, W-statistic=16.0<br>Rise time mean of spikes: p=0.0002, W-statistic=0.0<br>Latency first spike: p=0.7868, W-statistic=40.5<br>ISI mean of spikes: p=0.0681, W-statistic=19.0

Supplemental Table 2: *p*-values for action potential properties in early depolarisation, related to Figure 2.

Property	All MSNs		D1-MSNs		D2-MSNs	
	First spike	Spike train mean	First spike	Spike train mean	First spike	Spike train mean
Threshold (mV)	0.023 (↓)	0.002 (↓)	0.23	0.009 (↓)	0.026 (↓)	0.227
Height (mV)	0.029 (↓)	0.242	0.037 (↓)	0.995	0.600	0.010 (↓)
AHP (mV)	0.0005 (↓)	0.060	0.004 (↑)	0.008 (↓)	0.104	0.770
Width (ms)	1.684×10^{-9} (↓)	2.274×10^{-13} (↓)	2.726×10^{-9} (↓)	7.451×10^{-9} (↓)	0.008 (↓)	6.104×10^{-5} (↓)
Rise Time (ms)	1.137×10^{-13} (↓)	1.730×10^{-10} (↓)	3.725×10^{-9} (↓)	5.215×10^{-7} (↓)	3.725×10^{-9} (↓)	0.008 (↓)
Latency (ms)	0.005 (↓)	N/A	0.046 (↓)	N/A	0.030 (↓)	N/A
ISI (ms)	N/A	0.002 (↑)	N/A	0.012 (↑)	N/A	0.17

Supplemental Table 3: *p*-values for action potential properties in late depolarisation, related to Figure 2.

Property	All MSNs		D1-MSNs		D2-MSNs	
	First spike	Spike train mean	First spike	Spike train mean	First spike	Spike train mean
Threshold (mV)	0.042 (↑)	0.0002 (↓)	0.236	0.010 (↓)	0.168	0.013 (↓)
Height (mV)	0.569	3.275×10^{-8} (↑)	0.236	3.180×10^{-5} (↑)	0.635	0.0005 (↑)
AHP (mV)	0.0002 (↓)	0.552	0.010 (↑)	0.937	0.027 (↓)	0.340 (↑)
Width (ms)	$4,362 \times 10^{-6}$ (↓)	4.547×10^{-13} (↓)	4.212×10^{-5} (↓)	7.451×10^{-9} (↓)	0.055	0.0002 (↓)
Rise Time (ms)	0.002 (↓)	1.955×10^{-11} (↓)	0.002 (↓)	2.459×10^{-7} (↓)	0.433	0.0002 (↓)
Latency (ms)	0.010 (↓)	N/A	0.009 (↓)	N/A	0.787	N/A
ISI (ms)	N/A	0.0005 (↓)	N/A	0.008 (↓)	N/A	0.070

Supplemental Table 4: Amplitude of optogenetically evoked excitatory responses and steady-state measurement of optogenetically evoked inhibition measured from pre-stimulus baseline, related to Figure 3.

Response Group	First peak amplitude (mV, mean±SEM)	All peaks amplitude (mV, mean±SEM)	Steady-state inhibition (mV, mean±SEM)
1 Hz excitatory	8.973±2.066	9.350±1.018	N/A
5 Hz excitatory	10.032±2.857	6.072±0.756	N/A
10 Hz excitatory	7.335±1.492	4.184±0.403	N/A
20 Hz excitatory	7.478 ±1.319	2.033±0.010	N/A
10 ms excitatory	10.211±1.602	N/A	N/A
500 ms excitatory	21.917±2.694	N/A	N/A
1 Hz excitatory/inhibitory	0.222±0.123	0.368±0.051	N/A
5 Hz excitatory/inhibitory	0.215±0.027	0.226±0.028	N/A
10 Hz excitatory/inhibitory	0.173±0.068	0.097±0.016	N/A
20 Hz excitatory/inhibitory	0.421±0.106	0.010±0.005	N/A
10 ms excitatory/inhibitory	0.227±0.029	N/A	N/A
500 ms excitatory/inhibitory	8.044±1.393	N/A	N/A
10 ms excitatory/inhibitory	N/A	N/A	-0.118±0.033
500 ms excitatory/inhibitory	N/A	N/A	-0.994±0.075
500 ms inhibitory	N/A	N/A	-0.973±0.076
